# Cellular Responses Modulated by FGF-2 Adsorbed on Albumin/Heparin Layer-by-Layer Assemblies

**DOI:** 10.1371/journal.pone.0125484

**Published:** 2015-05-06

**Authors:** Marta Kumorek, Dana Kubies, Elena Filová, Milan Houska, Naresh Kasoju, Eliška Mázl Chánová, Roman Matějka, Markéta Krýslová, Lucie Bačáková, František Rypáček

**Affiliations:** 1 Department of Biomaterials and Bioanalogous Systems, Institute of Macromolecular Chemistry, Academy of Sciences of the Czech Republic v.v.i., Prague, Czech Republic; 2 Department of Biomaterials and Tissue Engineering, Institute of Physiology, Academy of Sciences of the Czech Republic, v.v.i., Prague, Czech Republic; 3 Department of Medical Biophysics and Informatics, Third Faculty of Medicine, Charles University in Prague, Prague, Czech Republic; 4 Department of Biomedical Technology, Faculty of Biomedical Engineering, Czech Technical University in Prague, Kladno, Czech Republic; Chang Gung University, TAIWAN

## Abstract

In a typical cell culture system, growth factors immobilized on the cell culture surfaces can serve as a reservoir of bio-signaling molecules, without the need to supplement them additionally into the culture medium. In this paper, we report on the fabrication of albumin/heparin (Alb/Hep) assemblies for controlled binding of basic fibroblast growth factor (FGF-2). The surfaces were constructed by layer-by-layer adsorption of polyelectrolytes albumin and heparin and were subsequently stabilized by covalent crosslinking with glutaraldehyde. An analysis of the surface morphology by atomic force microscopy showed that two Alb/Hep bilayers are required to cover the surface of substrate. The formation of the Alb/Hep assemblies was monitored by the surface plasmon resonance (SPR), the infrared multiinternal reflection spectroscopy (FTIR MIRS) and UV/VIS spectroscopy. The adsorption of FGF-2 on the cross-linked Alb/Hep was followed by SPR. The results revealed that FGF-2 binds to the Alb/Hep assembly in a dose and time-dependent manner up to the surface concentration of 120 ng/cm^2^. The bioactivity of the adsorbed FGF-2 was assessed in experiments *in vitro*, using calf pulmonary arterial endothelial cells (CPAE). CPAE cells could attach and proliferate on Alb/Hep surfaces. The adsorbed FGF-2 was bioactive and stimulated both the proliferation and the differentiation of CPAE cells. The improvement was more pronounced at a lower FGF-2 surface concentration (30 ng/cm^2^) than on surfaces with a higher concentration of FGF-2 (120 ng/cm^2^).

## Introduction

In a typical cell culture system, systematic administration of growth factors in the culture medium is required to stimulate cell growth. Alternatively, direct immobilization of growth factors on the cell culture surfaces may provide a constant reservoir of bio-signaling molecules without the need for additional supplementation of the culture medium with growth factors. It has been shown[1] that such systems can enhance cell growth due to optimized local concentration of the growth factor, which results in stronger interactions with the cell membrane receptors. Properly designed substrates can stabilize the growth factor molecules, enhance activity and control their release. The surface can be modified with proteins by direct covalent attachment of the biomolecule to the support, or by physicochemical adsorption based on specific and/or non-specific interactions between the biomolecule and the surface.[2] Covalent immobilization can affect the stability and the bioactivity of biomolecules because of the random orientation of the bounded protein, or can lead to the loss of functional groups present on the protein surface due to coupling reactions. Covalent binding methods also show low selectivity, thus resulting in co-immobilization of the impurities present in the protein solution.[3, 4]

In some cases, it is desirable to attach the protein molecule to the surface by making use of its natural affinity to the specific biological substances. This strategy is commonly applied to adsorb basic fibroblast growth factor (FGF-2) on heparinized surfaces. Basic fibroblast growth factor plays an important role in the proliferation and differentiation processes of a wide range of cells. However, when injected *in vivo*, FGF-2 has a short half-life time and undergoes rapid denaturation. In order to protect FGF-2 from degradation and to prolong its bioactivity, FGF-2 is usually applied with heparin or other sulfated compounds.[5] Heparin interacts effectively with FGF-2 and participates in the complex formation between FGF-2 and the cell surface FGF receptor [6–8], protects FGF-2 from inactivation and maintains the potency of FGF-2 even in harsh conditions.[9] On the other hand, albumin is also commonly used as a protein carrier including FGF-2, and is known to enhance the stability of FGF-2 and to prolong its shelf-time.[10, 11]

Heparin is a linear glycosaminoglycan containing numerous sulfo and carboxyl groups. As a strong polyanion, heparin has been used successfully in forming electrostatic layer-by-layer (eLbL) assembled capsules or films for growth factor delivery.[12–15] The eLbL technique is based on alternating electrostatic adsorption of polyelectrolytes with opposite charges on the surface yielding multilayer coatings of the nano- and micro-meter scale and can be easily applied for two-dimensional supports as well as for three-dimensional templates. The technique offers among other advantages (for review see ref. [16–18]), a control over the surface composition; the type of the terminal deposited layer of the coating can be easily tuned depending on the application. This technical aspect is used in growth factor delivery from eLbL films. A heparin/vascular endothelial growth factor (VEGF) eLbL coating on titanium surfaces improved endothelial cell proliferation and blood compatibility.[19] Cock et al.[13] incorporated transforming growth factor β1 (TGF-β1) into heparin/poly-L-arginine microcapsules for tissue engineering applications. FGF-2 adsorbed on chitosan/heparin films led to higher density of mesenchymal stem cells (MSCs) as compare to FGF-2 added into the culture media.[20] Cai et al.[21] showed that adsorbed bone morphogenetic protein 2 (BMP-2) on poly(allylamine hydrochloride)/poly(sodium 4-styrenesulfonate) eLbL film enhanced early osteogenic differentiation of MSCs. However, the selection of a polyanion/polycation pair plays an important aspect of the design of eLbL film for biological applications. For example, some of polycations, e.g. polyallylamine hydrochloride or poly(ethylenimine), can be cytotoxic after release into the culture media.[22, 23]

**Fig 1:**
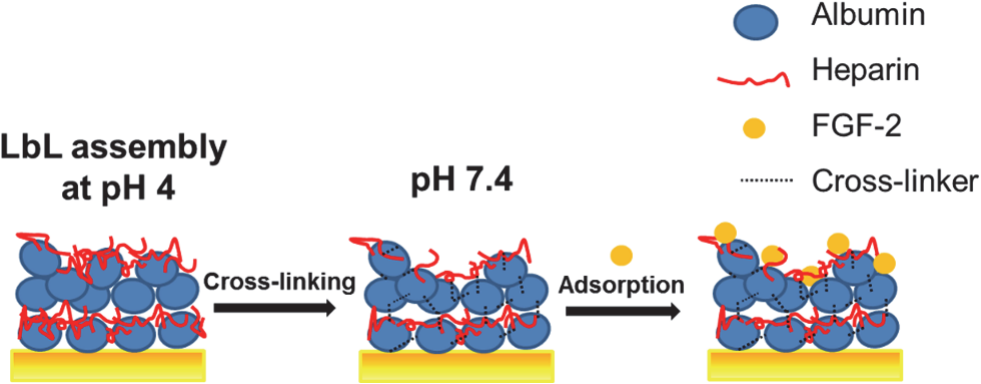
Schematic illustration of the formation of albumin/heparin assembly with adsorbed FGF-2. Step 1: eLbL assembly of (Alb/Hep)_2_ in a citrate buffer (pH 4); step 2: cross-linking of (Alb/Hep)_2_ assembly with glutaraldehyde and pH reversal to PBS buffer (pH 7.4); step 3: adsorption of FGF-2 on the cross-linked (Alb/Hep)_2_.

Obviously, the properly designed eLbL coating can capture the bioactive molecules via specific non-covalent interactions, while enhancing and preserving their bioactivity and facilitating their local delivery to the cells. By controlling the concentration of bioactive molecules in the eLbL film it is possible to manipulate the early cellular processes such as cell adhesion as well as the later one like proliferation, differentiation and tissue formation.[24–27] This paper follows on from our earlier works [28, 29] on the preparation and properties of multilayer eLbL assemblies of albumin and heparin (Alb/Hep), originally designed to improve materials for blood-contacting medical devices. Albumin and heparin, two abundant and naturally occurring compounds, are known from their ability to bind and protect FGF-2 from inactivation. Furthermore albumin, which serves as a primary anchoring layer to the substrate, can adsorb easily and strongly on a wide range of surfaces. This is in contrast to other types of polyelectrolytes used in eLbL technique that require a presence of a stable charge on the surfaces to initiate the eLbL self-assembly process. At physiological pH, albumin bears a net negative charge, but it is positively charged below its isoelectric point 4.9.[30] Here, we adsorbed albumin (Alb) on the surface at pH 4 and then adsorption of heparin (Hep) at pH 4 followed. These steps can be repeated until the required number of bilayers is deposited. Before pH reversal to neutral pH, the Alb/Hep system is fixed by cross-linking with glutaraldehyde. In contrast to other studies where both eLbL components are mutually attracted (e.g. heparin/chitosan, poly(L-lysine)/hyaluronan), heparin in the cross-linked Alb/Hep assembly is no longer involved in interactions with its counterpart that consume its charges, and is thus better available for the subsequent interaction with FGF-2. The model structure of the coating is depicted in Fig 1. Using SPR, FTIR and UV/Vis analysis, we proved the formation of the Alb/Hep assembly and demonstrated that the cross-linked Alb/Hep assembly can bind FGF-2 in a dose- and time-dependent manner. The bioactivity of the adsorbed FGF-2 and the overall cellular responses to the Alb/Hep assembly loaded with FGF-2 were verified and studied in *in vitro* experiments, using calf pulmonary arterial endothelial cells (CPAE) as a model cell line.

## Experimental Section

### 1. Materials

Recombinant human basic fibroblast growth factor (FGF-2) 146 aa (carrier free, purity better than 97%, lyophilized) was purchased from R&D Systems (Minneapolis, USA). Just before use, it was reconstituted in PBS containing 0.1% of human serum albumin. Human serum albumin (≥97%) and heparin sodium salt from porcine intestinal mucosa (17–19 kDa), glutaraldehyde (25% aqueous solution), (3-aminopropyl)triethoxysilane (APTES, 99%), fluorescein isothiocyanate isomer I (FITC, ≥90%) were obtained from Sigma-Aldrich. All the salts and organic solvents were of analytical grade. Deionized water (18.2 MΩ, Milli-Q Ultrapure Water System, Millipore) was used in all experiments.

Albumin was conjugated with FITC according to the published protocol[31] with small modifications. Briefly, 20 mg of Alb was dissolved in 0.1 M carbonate-bicarbonate buffer (pH 9) to a final concentration of 2.5 mg/mL. The FITC solution (9.5 mg/mL) was added drop-wise to the albumin solution, while stirring. The reaction was carried out 4 h in the dark at room temperature. The product was dialyzed (Spectrapor, cut-off 3.5 kDa) against water. Finally, the Alb^FITC^ product was lyophilized and the degree of labelling was determined spectroscopically according to the protocol suggested by the supplier (Sigma Aldrich). The molar ratio of FITC to Alb was 0.7.

### 2. Preparation of albumin/heparin coatings

The albumin/heparin (Alb/Hep) coatings were prepared on the gold surface of the SPR chips, on the ZnSe reflection element for FTIR MIRS analysis, on the silanized quartz slide for UV/Vis measurement and on polystyrene tissue culture plates (TCPS) for AFM analysis and for *in vitro* studies. The procedure was essentially the same, differing mainly in the pretreatment of the substrates.

#### 2.1. Coating of SPR chips

The formation of albumin/heparin coating and its interaction with FGF-2 was monitored *in situ* by SPR spectroscopy, using a custom-built SPR sensor (Institute of Photonics and Electronics, Academy of Sciences of the Czech Republic) with the Kretschmann configuration and wavelength interrogation detection mode. The adsorption was monitored in real time as a shift in the resonant wavelength. The SPR chip was rinsed with ethanol and water, dried with nitrogen and cleaned with a UV-ozone cleaner (Jelight) for 20 min before use. The chip was placed in a flow cell chamber of the SPR instrument and the system was primed with citrate buffer (CB, pH 4) for 5 min. CB was then replaced (purged out) with a solution of 1 mg albumin/mL in CB at a flow rate of 25 μL/min for 40 min. Then the albumin solution was replaced by CB for 5 min and after that the solution of 1 mg heparin/mL in CB was pumped in for 20 min. The solution of heparin was replaced by CB and the whole procedure, starting with the albumin solution, was performed once more. After the second Alb/Hep bilayer was deposited, it was cross-linked with 1% glutaraldehyde solution in CB for 30 min. Afterwards the whole system was rinsed with CB for 5 min and then the CB was replaced by PBS pH 7.4. To speed up the preparation of a bigger batch of coated chips for the adsorption experiments with FGF-2, the Alb/Hep-coated chips were prepared outside the SPR instrument. In this case, the final cross-linking was performed at a temperature of 55°C.

The detected wavelength shift was used to calculate the surface coverage. For the SPR sensor that was used, a shift of 1 nm represents surface coverage of 15 ng/cm^2^.[32] The detection limit of the SPR measurements (3×SD of the baseline noise) corresponds to surface coverage of 0.1 ng/cm^2^.

#### 2.2. Coating of a ZnSe reflection element

The infrared multiinternal reflection spectroscopy (FTIR MIRS) was used to monitor *in situ* the buildup process of albumin/heparin layers on the ZnSe reflection element (SPT, 458, Harrick Sci. EM2121). The surface of the ZnSe substrate was hydrophobized prior to use by spin coating with a polystyrene layer (PS). The PS-coated ZnSe was placed in the flow cell chamber of the FTIR spectrometer Bruker IFS55. The albumin and heparin were deposited on the PS-coated ZnSe and cross-linked according to the procedure described above (2.1.). After replacement of each solution, the FTIR spectra was recorded and changes of the characteristic infrared bands of both components (albumin: 1549 cm^-1^; heparin: 1030 cm^-1^) were examined.

#### 2.3. Coating of silanized quartz slides

The quartz slides were washed by sonication in petroleum ethyl ether and methanol, dried in a vacuum oven and exposed to air plasma (Harrick, Plasma cleaner/sterilizer) for 10 min. The cleaned quartz slides were silanized by APTES according to Krishnan et al.[33] The hydrophobized quartz slide was coated with fluorescently labelled albumin (Alb^FITC^) and Hep layers using the procedure described in the section 2.1. Alb was fluorescently labelled with FITC (Alb^FITC^) to enhance absorbance at 280 nm. After each Alb^FITC^/Hep bilayer deposition, the UV/Vis absorption spectrum (Specord Plus, Analytik Jena) in the range of 200–550 nm was measured.

#### 2.4. Coating the polystyrene tissue culture dishes (TCPS)

The procedure was by analogy with the procedure described for the SPR chips, with minor deviations. The wells of the sterile 24-well tissue culture plates TCPS (Multitwell, Becton Dickinson Labware) were incubated for 1 h with 2 mL of the albumin solution (1 mg/mL in CB), rinsed 3 times with CB, then incubated for 30 min with 2 mL of heparin solution and rinsed 3 times with CB. These steps were repeated until two albumin/heparin bilayers (denoted in the text as (Alb/Hep)_2_) were deposited. Finally, the (Alb/Hep)_2_ assembly was cross-linked with 1 mL of 1% glutaraldehyde (in CB, pH 4) for 1 h at 55°C, rinsed 3 times with CB followed by PBS. The procedure was performed under sterile conditions in a cell-culture flow-box.

### 3. Interaction of the albumin/heparin coating with FGF-2

The loading of the (Alb/Hep)_2_ coating with FGF-2 was observed using SPR spectroscopy analysis. FGF-2 in a concentration of 500 ng/mL or 1000 ng/mL in a co-solution with 0.1% solution of albumin in PBS was pumped at flow rate of 25 μl/min. After a given time, the FGF-2 co-solution was replaced by PBS and the stability of the complex was monitored for approximately 13 h.

### 4. AFM measurement

Atomic force microscopy of the Alb/Hep assemblies were performed on Dimension Icon (Bruker) in Tapping mode in a buffer environment. The 256 × 256 pixel topography images were acquired using sharp microlever probes (MSNL, Bruker) with cantilever resonance frequency about 60 kHz, cantilever spring constant 0.1 N/m and scan rates in the range of 0.55 to 0.8 Hz. The topography images of reference surface, i.e. TCPS dish (TPP, Switzerland) were acquired under similar condition in contact mode. NanoScope Analysis software (Bruker) was used for data processing.

### 5. Cell culture studies

#### 5.1. Surface preparation: Adsorption of FGF-2 on the Alb/Hep coating

The TCPS wells coated with (Alb/Hep)_2_ bilayers (see paragraph 2.4) were incubated with a solution of FGF-2 in 0.1% albumin in PBS. Two concentrations of FGF-2 were used: 500 ng/mL and 1000 ng/mL. 300 μL of the FGF-2 solution was added into each well, and was allowed to adsorb for 3 h (in the case of 1000 ng/mL FGF-2) or for 1 h (in the case of 500 ng/mL FGF-2) under agitation (50 rpm). Then, the protein solution was aspirated off, and the wells were washed 3 times with PBS. All plates were sterilized by irradiation with a UV lamp for 30 min prior to cell seeding.

#### 5.2. Cell culture

The endothelial cells, (CPAE line, ATCC CCL-209, Rockville, MA, U.S.A) were seeded onto 24-well TCPS uncoated or coated with (Alb/Hep)_2_ assembly at a density of 30 000 cell/well and cultured at 37°C in a humidified atmosphere of 5% CO2 and 95% air incubator. The cell culture medium consisted of Minimum Essential Eagle Medium with 2 mM L-glutamin, Earle’s BSS with 1.5 g/L sodium bicarbonate, 0.1 mM non-essential amino acids, 1.0 mM sodium pyruvate and 5% of fetal calf serum (FBS, Cat. No. F7524, Sigma). The cells were cultured for 6 or 7 days.

#### 5.3. Determination of the optimal dose of FGF-2 in the culture medium

The cells were seeded onto 24-well TCPS plate coated with the (Alb/Hep)_2_ assembly. The cells were cultured in 1.5 mL of the cell culture medium mentioned above, supplemented with 5% of fetal calf serum and 0, 0.1, 1, 10, and 100 ng/mL of FGF-2. Images of the living CPAE cells were taken using an Olympus IX 71 epifluorescence microscope and a DP 71 digital camera (Japan). The cells were counted on day 1, 3, and 6 after initial seeding from 20–30 micrographs, and the cell densities were calculated.

#### 5.4. Cell morphology, number and growth curves

On day 1, the cells on the tested surfaces were fixed in 2% paraformaldehyde in PBS for 5 min and stained with Texas Red C2 maleimide (1 ng/mL, 1 h, Life Technologies) and Hoechst 33342 (5 μg/mL, 10 min). The cell morphology and the cell number were assessed either from micrographs of the stained cells or from pictures of the living cells without staining (on the surfaces with FGF-2 in medium). The number of cells was evaluated from 20–23 micrographs taken under an epifluorescence microscope (Olympus IX71, digital camera DP71, Japan). On day 3 and 7, the cells on the samples were harvested with a trypsin-EDTA solution (Sigma, U.S.A, Cat. No. T4174) in PBS, and their number was measured using a Cell Viability Analyzer (VI-cell XR, Beckman Coulter, 3–6 measurements for each experimental group). The cell viability was also measured using a trypan blue exclusion test in the Cell Viability analyzer. Cell densities per cm^2^ were used for constructing the growth curves and for calculating the cell population doubling time on the tested surfaces.

#### 5.5. Cell spreading area and doubling time

The spreading area of CPAE cells was determined on day 1 from 165–550 cells on 11 randomly chosen regions for each surface using Atlas software (Tescan Ltd., Czech Republic). The cell population doubling time (DT) between days 1 and 3 or 3 and 6 was calculated from 17–66 measurements on the basis of the equation:

$$DT=\frac{\log2\times dt}{\log(N)-\log(N_{0})}$$

Where *dt* stands for time of cultivation in hours, *N* and *N*_*0*_ correspond to the cell number on day 3 (or 6) and day 1 (or 3) after seeding, respectively.

#### 5.6. Immunofluorescence staining of vinculin, BrdU, von Willebrand factor, and VE-cadherin

On day 3 (vinculin, BrdU), day 6 (von Willebrand factor for evaluating the optimal dose of FGF-2 in the culture medium), or day 7 (von Willebrand factor, VE-cadherin), the cells were fixed in 2% paraformaldehyde in PBS for 5 min (pH 7.4), washed three times in PBS (5 min), pre-treated with 1% bovine serum albumin in PBS containing 0.1% Triton X-100 (1 h) and then incubated in 1% Tween 20 for 20 min. After 5 min washing in PBS, the primary antibodies, i.e. monoclonal anti-vinculin, mouse ascites fluid, clone hVIN-1 (Sigma, Cat. No. V 9131, dilution 1:100 in PBS), polyclonal rabbit anti-human CD144 (VE-cadherin), purified IgG (AbD Serotec, UK, Cat. No. AHP628Z, dilution 1:200), or rabbit anti-human von Willebrand factor (Sigma, Cat. No. F3520, dilution 1:200) were applied overnight at 4°C. As the secondary antibodies, Alexa Fluor 488-conjugated F(ab’) fragment of goat anti-mouse IgG (H+L) or Alexa Fluor 488-conjugated F(ab’) fragment of goat anti-rabbit IgG (H+L), or Alexa Fluor 546-conjugated F(ab’) fragment of goat anti-rabbit IgG (H+L), (Molecular Probes, Cat. No. A11017, A11070 or A11010, respectively, dilution 1:400) were used for 80 min at room temperature. 0.05% Tween 20 in PBS was used to wash the samples. The cell nuclei were counterstained with Hoechst 33342 (5 μg/mL, 10 min). The cells were evaluated under an epifluorescence microscope (Olympus IX71, digital camera DP71, Japan). In order to measure the fluorescence intensity, 10–11 micrographs from each sample were taken at the same exposure time on day 3 or 7 after seeding. The fluorescence intensity was measured using Fluorescence Image Analyser software (ver. 1.1, 2013, available from http://alice.fbmi.cvut.cz/software/fia). A single color plane threshold was set on each image to remove the non-protein area from the image data. These threshold and color plane settings were the same for each image of the protein that was measured. Then the cumulative sum of all pixel intensities was evaluated and the background intensity of the negative staining control was subtracted. The total immunofluorescence intensity of the protein was normalized to the number of cells in the microscopic field and expressed in values relative to (Alb/Hep)_2_. To evaluate the BrdU labelling index, the cells were counterstained with Texas Red C2 maleimide (1 ng/mL, 1 h, Life Technologies). The percentage of BrdU+ cells was analyzed on 14–24 microscopic fields of each sample.

#### 5.7. Statistical analysis

The quantitative data was presented as mean ± SEM (Standard Error of Mean). The statistical analyses were performed using SigmaStat (Jandel Corporation, USA). Multiple comparison procedures were made by the ANOVA, Student-Newman-Keuls method or by Kruskal-Wallis One Way Analysis of Variance on Ranks. The value p ≤ 0.05 was considered significant.

## Results and Discussion

### Albumin/heparin coatings

**Fig 2:**
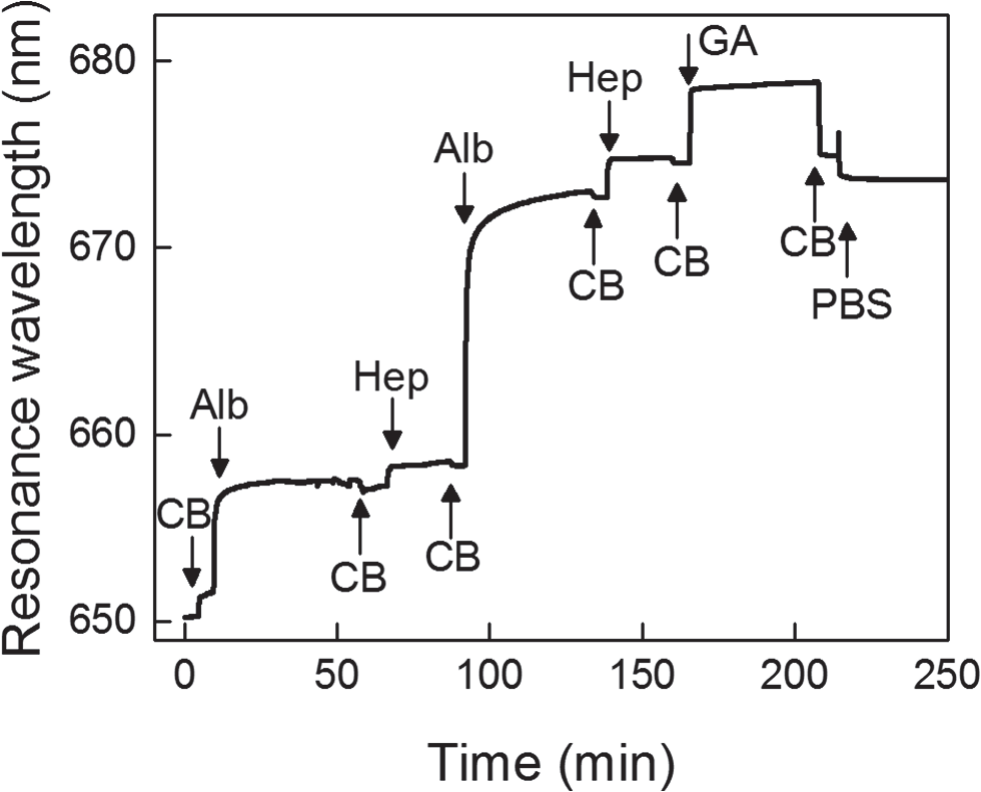
The adsorption of albumin (Alb) and heparin (Hep) layers and cross-linking with glutaraldehyde on a gold surface observed by SPR. The Alb and Hep was adsorbed from 1 mg/mL solutions in a citrate buffer (CB, pH 4); phosphate buffer saline (PBS, pH 7.4), glutaraldehyde (1% GA in CB). Arrows indicate replacement of the solutions.

A typical SPR sensogram of the formation of the Alb/Hep coating is shown in Fig 2. During the deposition of the particular layer, the measured signal (a shift in resonant wavelength) increases as a result of the increase of the refractive index at the interface. A decrease in the measured signal is observed during the rinsing step, when the unbound part of the albumin or heparin is removed from the surface.

It is generally accepted that a change in the refractive index corresponds to changes in the mass of the adsorbed biomolecule, and is the same for all proteins and peptides.[34–36] Based on previous work,[32] a shift in the resonant wavelength equal to 1 nm corresponds to 15 ng of biomolecule adsorbed at 1 cm^2^. Using the conversion rate of the resonant frequency shift to the mass coverage, about 110 ng of albumin/cm^2^ was adsorbed in the first irreversible layer after 40 min of deposition (Fig 2). The adsorption of the primary albumin layer is driven mainly by hydrophobic interactions. The determined value of the adsorbed albumin is in good agreement with our previous results,[28] and corresponds to the formation of a monomolecular protein layer. After replacement of the albumin solution by the citrate buffer (CB), the albumin layer was subjected to the heparin solution and after 20 minutes about 20 ng/cm^2^ of heparin was adsorbed. Here we assume that no albumin is resolubilized (removed from the surface) in the form of a soluble albumin/heparin complex, as was confirmed by infrared spectroscopy in our previous work.[28, 37] After replacement of the heparin solution by CB, albumin was again introduced. Now, the driving force was the electrostatic interaction with the overcharged primary albumin/heparin bilayer and about 260 ng of albumin/cm^2^. The electrostatic interactions thus led to more than twice as much albumin adsorbed as in the first albumin layer formed by hydrophobic interactions. After rinsing with CB, the heparin solution was introduced and about 45 ng/cm^2^ of heparin was adsorbed. Since this eLbL system is held together only by electrostatic forces at pH 4, covalent cross-linking with glutaraldehyde (GA) in CB was performed before the transfer of eLbLs to the physiological pH. Without cross-linking, only the primary albumin layer held by hydrophobic interactions with the SPR chip would be retained on the surface.[38] To observe the overall build-up process, the cross-linking reaction was carried out *in situ* at 25°C directly in a flow cell of the SPR instrument. The fixation process can, however, be improved when the cross-linking is performed at 55°C outside the SPR apparatus. Fig 2 shows only a minor decrease in the mass of the (Alb/Hep)_2_ deposit after the cross-linking procedure and a final rinse with PBS. SPR analysis does not enable us to determine which component desorbed, but the results presented in Fig 2 correlate very well with our previous findings[28] that the whole amount of albumin and approximately 50% of the heparin were retained on the surface. Based on these considerations, we can deduce that the final (Alb/Hep)_2_ assembly on gold contains about 370 ng of albumin and 30 ng of heparin/cm^2^. These amounts are slightly lower than the amounts found for the (Alb/Hep)_2_ bilayer on polystyrene. This is not surprising, as the final amounts are determined by the density of the primary albumin layer, which is higher when adsorbed on polystyrene; however, the albumin/heparin ratios are almost the same.[28]

**Fig 3:**
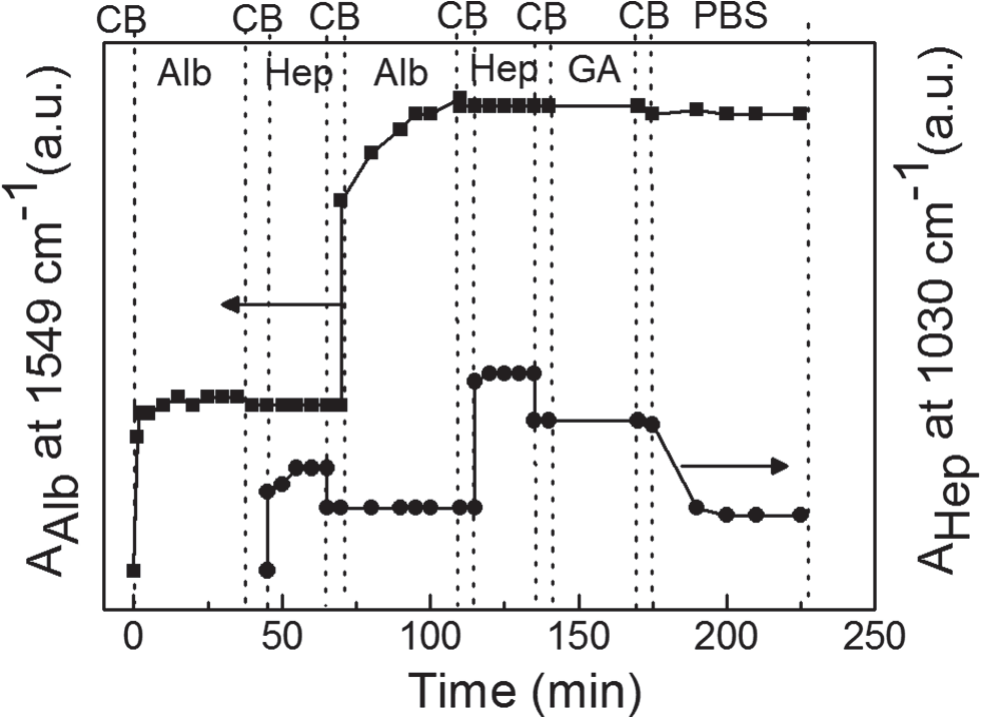
The adsorption of albumin (Alb) and heparin (Hep) layers and cross-linking with glutaraldehyde on a PS-coated ZnSe observed *in situ* by FTIR MIRS. The Alb (■) and Hep (●) was adsorbed from 1 mg/mL solutions in a citrate buffer (CB, pH 4); phosphate buffer saline (PBS, pH 7.4), glutaraldehyde (1% GA in CB).

Fig 3 presents FTIR MIRS *in situ* recording of a layer-by-layer assembling of Alb and Hep and subsequent cross-linking of the deposited coating. The analysis of FTIR MIRS data enabled a separate detection of both individual components during each adsorption step (S1 Fig). Similarly to SPR results, the amount of the adsorbed Alb and Hep in the second eLbL cycle was more than twice as compare to primary Alb and Hep layers. Fig 3 also shows that after cross-linking with GA and transfer to PBS the amount of Alb fixed in the assembly remained essentially unchanged while the amount of Hep fell down to about one half due to a less efficient cross-linking reaction (a low concentration of available amino groups in Hep chains). More detailed analysis of FTIR data was performed in our earlier works.[28, 29, 37, 39]

**Fig 4:**
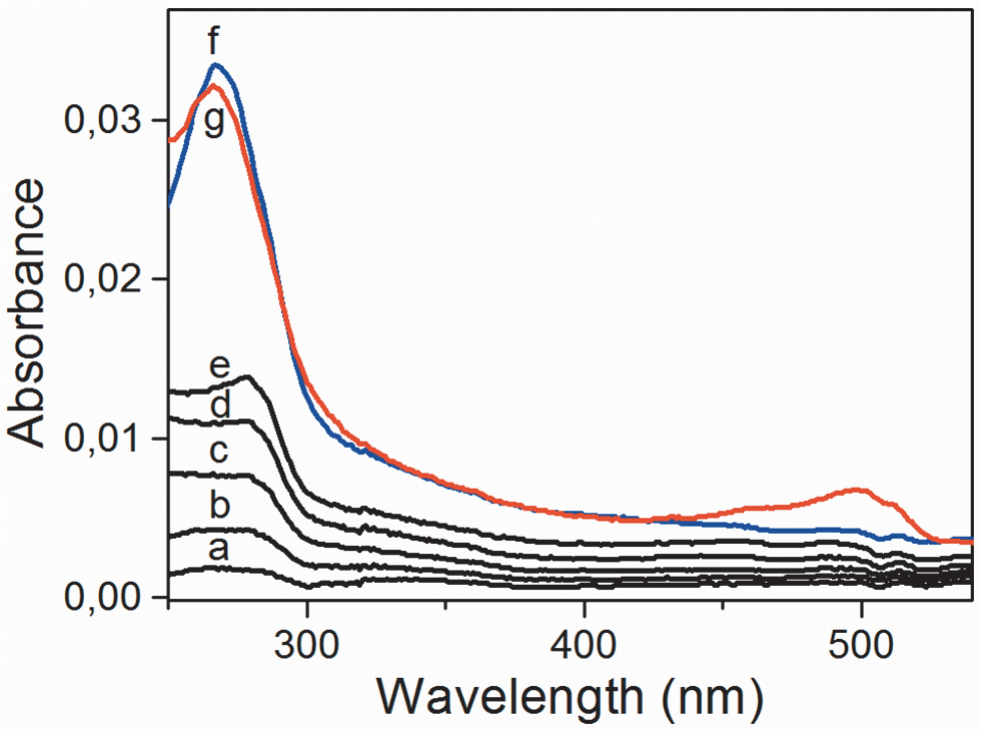
UV/Vis absorption spectra of Alb^FITC^/Hep films assembled on a silanized quartz slide with increasing bilayer numbers (a—e). The Alb^**FITC**^ and Hep was adsorbed from 1 mg/mL solutions in a citrate buffer (CB, pH 4; a—e). The (Alb^**FITC**^/Hep)_5_ assembly cross-linked with 1% glutaraldehyde (1% GA in CB; f) and transferred to phosphate buffer saline (PBS, pH 7.4; g).

The alternate adsorption of Alb and Hep during the eLbL process in a citrate buffer at pH 4 was monitored also by UV/Vis spectroscopy. The ability of albumin to absorb in UV region was enhanced by conjugation of fluorescein (FITC) to albumin. Fig 4 (plots a—e) shows the UV/Vis absorption spectra of 1, 2, 3, 4 and 5 bilayers of Alb^FITC^/Hep. The absorption band of albumin at 280 nm increased with an increasing number of deposited bilayers showing that eLbL Alb^FITC^/Hep assembly was successfully formed. Notably, a new absorption peak at 266 nm appeared in the spectra after cross-linking of the (Alb^FITC^/Hep)_5_ assembly with GA (Fig 4, plot f). According to the literature, a lot of chemical reactions are involved into the GA cross-linking mechanism and different chemical structures contributing to stable cross-links are formed.[40] We have attributed the peak at 266 nm to pyridinium derivatives formed by reaction of GA with amine groups of both Alb and Hep molecules.[41] Importantly, the peak remains almost unchanged when the cross-linked (Alb^FITC^/Hep)_5_ assembly is transferred from pH 4 to pH 7.4 (PBS) what indicates the proper fixation of the film (Fig 4, plot g). The new band at 495 nm in UV/Vis spectra at neutral pH was attributed to the fluorescein marker. It is well known that absorption properties of fluorescein are strongly pH dependent.[42] At pH 4, neutral species of fluorescein have a weak absorption in visible region whereas at pH 7.4 dianionic forms of fluorescein with the high molar excitation coefficient (77 000 M^-1^cm^-1^) at wavelength of 490 nm [42] dominate over other proteolytic forms. This explains why the band in visible region was not observed during Alb^FITC^/Hep deposition at pH 4 but was visible after pH replacing to 7.4.

Although the principle of the eLbL technique is described as an alternating deposition of polycations (here albumin) and polyanions (here heparin), the layers are, of course, not strictly geometrically separated and both albumin and heparin can be exposed on the surface (Fig 1). However, when considering (Alb/Hep)_2_ assembly, the terminal heparin prevails in the outermost layer as it is evidenced by the fact that an additional positively charged albumin can be further adsorbed on it (Figs 2, 3 and 4). Moreover, the results from X-ray photoelectron spectroscopy measurements performed in our earlier work[39] showed prevailing intensity of the sulfur or nitrogen signal in the upper layer of the coating when the film was terminated with the heparin or albumin layer, respectively. It indicates that the terminal layer in the presented cross-linked (Alb/Hep)_2_ film contains mostly heparin.

The morphological features of the eLbL Alb/Hep assemblies were examined by atomic force microscopy (AFM) in a swollen state in PBS (pH 7.4). The control TCPS well plate exhibited a unique surface topography with the visible fiber-like and circular-shape features (S2 Fig) which were already reported in the literature.[43] These surface defects of TCPS were also observed when substrate was coated by cross-linked (Alb/Hep)_1_ assembly (S2 Fig). Moreover, the AFM image taken at higher magnification revealed that the (Alb/Hep)_1_ film suffered from a lack of uniformity of the surface topography (S2 Fig, right column). On the contrary, more homogenous and a fine surface structure was found for the cross-linked (Alb/Hep)_2_ and (Alb/Hep)_3_ assemblies (S2 Fig). Furthermore, the AFM results showed that surface morphology of (Alb/Hep)_2_ assembly at pH 4 did not changed after film cross-linking and transferring to pH 7.4 (S3 Fig).

### Adsorption of FGF-2 on the (Alb/Hep)_2_ coating

**Fig 5:**
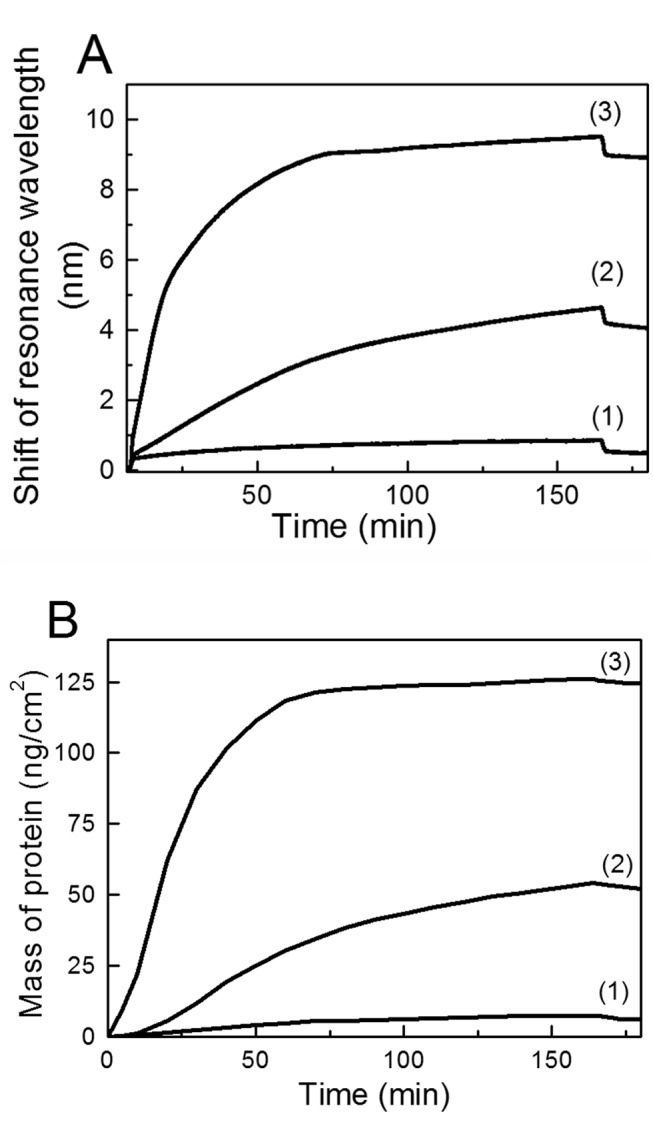
Adsorption of FGF-2 and albumin on (Alb/Hep)_2_ film. (**A**) SPR sensogram and (**B**) time dependent mass adsorption of (1) 0.1% albumin, (2) 500 ng/mL FGF-2 and (3) 1000 ng/mL FGF-2 to (Alb/Hep)_2_. The FGF-2 solutions contained 0.1% albumin.

Adsorption of FGF-2 was performed on two albumin/heparin bilayers ((Alb/Hep)_2_), i.e., the minimum number of layers identified to be able to screen off the effect of the underlying substrate (S2 Fig). Fig 5A presents the SPR sensograms of the adsorption of albumin and FGF-2 at two concentrations, i.e. at 500 ng/mL and at 1000 ng/mL. All curves show small jump shifts following replacements of the solutions which reflects differences between the biomolecule solution and PBS. Since the FGF-2 deposition was performed in PBS containing 0.1% of albumin, we first verified whether the (Alb/Hep)_2_ coating can adsorb albumin from a solution in PBS. The SPR chip coated with the (Alb/Hep)_2_ bilayer was equilibrated in PBS, then PBS was replaced by a 0.1% solution of albumin in PBS, and after 160 min PBS was again introduced (Fig 5A, Curve 1). The results show a very small interaction of the cross-linked (Alb/Hep)_2_ bilayer with albumin. It was calculated that about 7 ng of albumin/cm^2^ was adsorbed, which is less than 2% of the total amount of albumin in the (Alb/Hep)_2_ coating (Fig 5B, Curve 1). Curves 2 and 3 of Fig 5A illustrate the FGF-2 adsorption process from the 500 ng/mL and 1000 ng/mL FGF-2 solutions. Curve 2 shows substantially slower adsorption from the 500 ng/mL FGF-2 solution without reaching apparent saturation in the course of 160 min, while Curve 3 depicted faster adsorption from the 1000 ng/mL FGF-2 solution slowed down after about 80 min and suggesting saturation after 160 minutes. Replacement of the FGF-2 solution by PBS after 160 min and further incubation in PBS leads to only minor changes indicating the stability of the (Alb/Hep)_2_ complex with FGF-2. On the basis of previous work,[32] we calculated the amount of FGF-2 adsorbed on (Alb/Hep)_2_ (Fig 5B). Curves 2 and 3 of Fig 5B show the changes in the amount of adsorbed FGF-2 within the time as adsorption proceeds. It was calculated that about 120 ng of FGF-2/cm^2^ is retained when surface saturation is reached (Fig 5B, Curve 3). Taking into account that the coating contains 30 ng heparin/cm^2^ and the molecular weights of the heparin and FGF-2 used are approximately the same, the calculated value corresponds to a ratio of about 4 molecules of FGF-2 to one molecule of heparin. This value is in accordance with the data found in the literature[8] reporting that the number of FGF-2 molecules incorporated into complex with the heparin was in the range of 1 to 6.

The short-term stability of the adsorbed FGF-2 (FGF-2_ads_) on (Alb/Hep)_2_ coatings in PBS at pH 7.4 was followed by SPR. S4 Fig (graph A) presents SPR sensograms of the FGF-2 adsorption on (Alb/Hep)_2_ assembly and a subsequent FGF-2 desorption into PBS. The initial surface concentration of FGF-2_ads_ was 30 ng/cm^2^ (S4 Fig, graph A, line a) or 120 ng/cm^2^ (S4 Fig, graph A, line b). A slight FGF-2_ads_ release occurred during 10 h after the growth factor loading; after this time the plateau was reached. The desorbed amount represents less than 2% or 6% of FGF-2_ads_ at concentration of 30 ng/cm^2^ or 120 ng/cm^2^, respectively (S4 Fig, graph B)_._ The small amounts released indicate the formation of a stable complex of FGF-2 with (Alb/Hep)_2._

Several approaches have been used to provide control over the content of growth factors within multilayered films. Macdonald et al.[44] studied the loading and release of FGF-2 from synthetic hydrolytically degradable multilayer thin films. The FGF-2 loading within the film was in the range of 7–45 ng/cm^2^, and was tuned by the number of polyelectrolyte layers and the type of polyanion (heparin sulfate or chondroitin sulfate) and polycation (degradable poly(β-aminoester)s). In another work,[12] eLbL assemblies were formed directly by using acid fibroblast growth factor (aFGF) and heparin. The amount of aFGF was controlled by the number of deposited layers. Crouzier et al.[24] showed that cross-linked poly(L-lysine)/hyaluronan films can be used for delivering bone morphogenetic protein 2 (rhBMP-2) in a dose-dependent manner. The amount of rhBMP-2 loaded into the eLbL films was modulated by varying the film thickness and the initial rhBMP-2 concentration in the suspending medium. In the present work, we show that FGF-2 can bind to the Alb/Hep assembly in a dose- and time-dependent manner. Fig 5B shows that the amount of FGF-2 adsorbed on the (Alb/Hep)_2_ assembly may be tuned up by the concentration of FGF-2 in the stock solution and/or by the time of adsorption. Within 160 min, FGF-2 adsorbed on the (Alb/Hep)_2_ assembly up to 45 ng/cm^2^ and 120 ng/cm^2^ from 500 ng/mL and 1000 ng/mL FGF-2 solutions, respectively.

**Fig 6:**
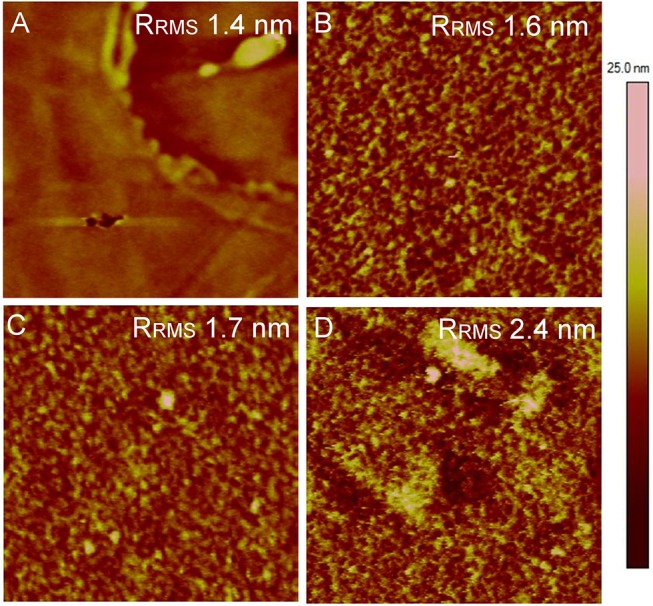
AFM topography images. (**A**) TCPS, (**B**) (Alb/Hep)_2_ assembly, (**C**) (Alb/Hep)_2_ with FGF-2_ads_ (30 ng/cm^**2**^), (**D**) (Alb/Hep)_2_ with FGF-2_ads_ (120 ng/cm^**2**^) in PBS. The root mean squared surface roughness (R_RMS_) is depicted in each image. Image size: 1 × 1 μm, Z-scale: 25 nm.

In addition, the effect of presence of FGF-2_ads_ on the (Alb/Hep)_2_ assembly on the film topography was examined. As can be observed in Fig 6C and 6D, the adsorption of FGF-2 on the (Alb/Hep)_2_ assembly did not alter the surface morphology. However, the presence of FGF-2_ads_ caused a slight increase in the root mean squared surface roughness (R_RMS_) of the film which was more prominent for a higher FGF-2_ads_ concentration (Fig 6B–6D). A similar surface morphology was observed for multilayer films formed by heparin and aFGF by Mao et al.[12]

### Adhesion, growth and differentiation of CPAE cells on (Alb/Hep)_2_ with soluble FGF-2 added into the cultivation medium (FGF-2_sol_)

First, we investigated the effect of soluble FGF-2 (FGF-2_sol_) added into the culture media on the adhesion, growth and differentiation of calf pulmonary arterial endothelial (CPAE) cells. The CPAE cells were cultured on TCPS coated with (Alb/Hep)_2_ and on the control TCPS surface in 5% FBS media. FGF-2 was added into the culture media to a final concentration of 0, 0.1, 1, 10 and 100 ng/mL, respectively.

**Fig 7:**
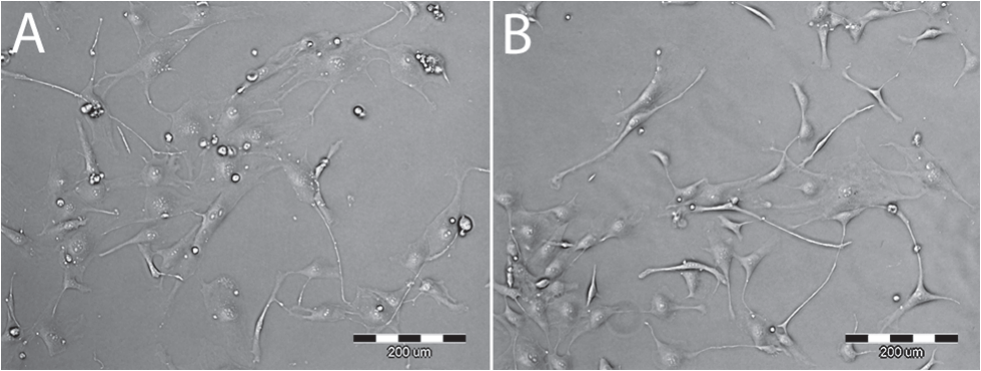
Images of living CPAE cells on TCPS (A) and (Alb/Hep)_2_ (B). The cells were cultivated in 5% FBS media. Images taken on day 3 after seeding. Obj. ×10, scale bar = 200 μm.

The morphology of the adhered CPAE cells was polygonal on TCPS and more elongated on the (Alb/Hep)_2_ surfaces (Fig 7). This observation is consistent with previously reported findings[45] that CPAE cells cultivated on soft and flexible fibrin gels displayed characteristic morphological features. The cells showed an elongated shape with the presence of protrusions, and the initial cell spreading area was smaller than on the TCPS control. This phenomenon was explained by the stiffness of the cultivation surface. The cell morphology observed on the (Alb/Hep)_2_ assembly could be also affected by a charge introduced on the substrate surface. Bone marrow derived mesenchymal stem cells and adipose stem cells seeded onto-SO_3_H functionalized surfaces showed star-like shapes and a high density net of filopodia and these morphological features were dependent on the—SO_3_H surface density.[46] Further, the presence of FGF-2 has also an impact on the appearance of the cells. The CPAE cells on the (Alb/Hep)_2_ surfaces with the presence of FGF-2_sol_ exhibited spindle-like morphologies and elongated shapes, and formed narrow, needle-shaped protrusions (S5 Fig). Similarly to our observation, supplementation of the culture medium with FGF-2 induced morphological changes of the endothelial cells, which became elongated, formed an irregular crisscross pattern and displayed fibroblast-like shapes.[47]

**Fig 8:**
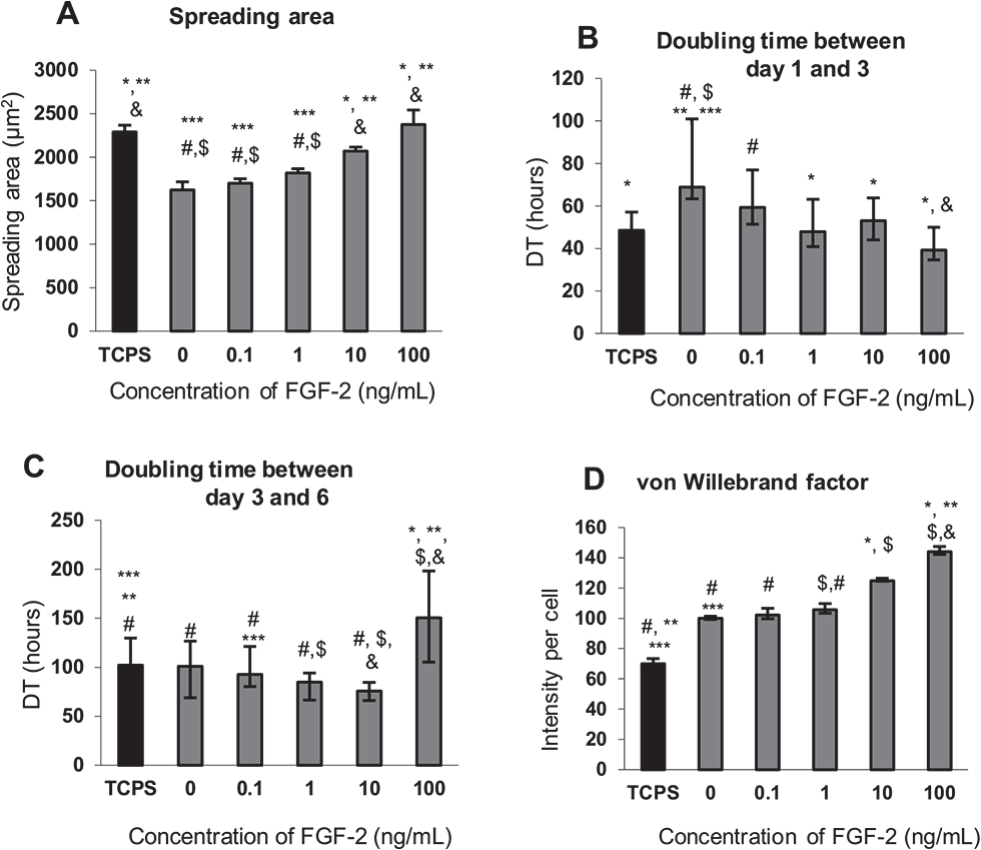
The effect of the amount of FGF-2_sol_ added into the cultivation media on the spreading area 24 h after seeding (A), doubling time between day one and three (B), doubling time between day three and six (C), and the intensity of immunofluorescence staining of von Willebrand factor (D) of CPAE cells cultivated on (Alb/Hep)_2_ surfaces and the control TCPS. The amount of FGF-2_sol_ added into the media was 0, 0.1, 1, 10 and 100 ng/mL. The cells were cultivated in 5% FBS media. The fluorescence intensity (D) is normalized per cell. The data is expressed as mean ± S.E.M (A) and median ± quartiles (B-D). Statistically significant differences (p < 0.05) are depicted above the bars in comparison with 0 ng/mL (*), 0.1 ng/mL (&), 1 ng/mL (**), 10 ng/mL (***), 100 ng/mL (#), and TCPS ($).

Importantly, the cell densities on day 1 were the same on all samples (S6 Fig). On the other hand, the spreading area depended on the FGF-2_sol_ dose (Fig 8A); the cells spread significantly better and comparably to TCPS only when the medium was supplemented with 10 and 100 ng/mL FGF-2_sol_. Generally, polysaccharide-based polyelectrolyte multilayer films do not support initial cell attachment. Mesenchymal stem cells (MSCs) poorly adhered to chitosan/heparin eLbL. The cell density on chitosan- and heparin- terminated eLbL was respectively 7.4 and 5.2 times lower in comparison with the TCPS control.[20] Similarly, the skeletal muscle cells cultured on poly(L-lysine)/hyaluronan multilayers showed 5 times lower cell density then on a plastic surface.[48] Mouse fibroblast cells (NIH/3T3) were unable to adhere to the FGF-2/heparin-functionalized thermoresponsive surface when the heparin density was above 1.6 μg/cm^2^,[49] which was explained by repulsion forces between negatively charged cell membranes and heparin chains. A heparin surface density providing good adhesive properties for NIH/3T3 cells was found to be 0.8 ± 0.4 μg/cm^2^. In our work, the amount of heparin in (Alb/Hep)_2_ used for further FGF-2 adsorption was 0.03 μg/cm^2^. Apparently, a relatively low amount of bound heparin was sufficient for CPAE cell attachment and proliferation during 7 days of culturing.

Divergent data can also be found in the literature related to the adhesive/anti-adhesive properties of albumin. It was shown that surfaces pre-coated with albumin did not support CPAE cells adhesion *in vitro*.[50] However, a marked increase in the adhesion of human endothelial cells (HUVEC) was observed on the commercial albumin-heparin conjugate covalently bound to a plasma CO_2_-treated polystyrene plate.[27] Due to the increased stiffness of the coating, the bovine endothelial cells also adhered and proliferated on the photopolymerized styrenated albumin, photopolymerized styrenated heparin and photo-copolymerized styrenated albumin and styrenated heparin layers.[51] Also polyelectrolyte polylysine/hyaluronic acid multilayers showed high resistance to cell adhesion, but the cells adhered and spread well after the film was cross-linked.[52] In our study, the (Alb/Hep)_2_ surface exhibited only approximately 10% lower initial cell attachment than the TCPS control (S6 Fig). This was likely due to increase in the rigidity of the Alb/Hep films as a result of cross-linking by glutaraldehyde.

On day 3, the CPAE cells on the (Alb/Hep)_2_ surface with no FGF-2_sol_ supplementation reached a significantly lower density than on the TCPS control (S6 Fig), and the doubling time between day 1 and 3 was also longer (Fig 8B). FGF-2_sol_ supplementation increased the cell densities to the levels on TCPS, and the positive effect of FGF-2_sol_ correlated proportionally with the FGF-2_sol_ concentration (S6 Fig). The addition of FGF-2_sol_ shortened the cell doubling time; cells proliferated better between day 3 and 6 on the (Alb/Hep)_2_ surfaces with added 1 and 10 ng/mL FGF-2_sol_, whereas 100 ng/mL FGF-2_sol_ significantly prolonged the cell doubling time (Fig 8C). On day 6, the CPAE cells reached similar densities on all surfaces with FGF-2_sol_ supplementation (S6 Fig). FGF-2_sol_ also stimulated the production of von Willebrand factor. This effect was dose-dependent (Fig 8D); the intensity of the fluorescence marker per cell was the highest on the (Alb/Hep)_2_ surface with 100 ng/mL of FGF-2_sol_ (Fig 8D and S7 Fig).

Different doses of FGF-2_sol_ in the cultivation media can induce different cellular responses. FGF-2 at a concentration of 5–20 ng/mL supported proliferation of human bone marrow mesenchymal stem cells,[53] whereas concentrations of FGF-2 higher than 40 ng/mL had no effect on cell growth. The behaviour of lens epithelial cells was also affected comprehensively by the presence of FGF-2.[54] The half maximal doses of FGF-2 in media required to induce proliferation, migration and fiber differentiation were 150 pg/mL, 3 ng/mL, and 40 ng/mL, respectively. At FGF-2 concentration higher by an order than the optimum concentration, all these cell functions were inhibited. Periodontal ligament cells were stimulated to proliferate until a concentration 10 ng/mL of FGF-2 in the cell culture media, whereas concentration higher than 100 ng/mL reduced the cell proliferation.[55] Our results correlate well with these data. Supplementation of the culture media with FGF-2_sol_ enhanced the CPAE cell proliferation on the (Alb/Hep)_2_ assemblies proportionally to the FGF-2_sol_ concentration in the concentration range 0.1–10 ng/mL (S6 Fig). A high dose of FGF-2_sol_ (100 ng/mL) tended to support cell spreading and cell division in the early stage of cultivation, but the proliferation started to decrease on day 6 and the CPAE cells were stimulated to differentiation.

### Adhesion, growth and differentiation of CPAE cells on (Alb/Hep)_2_ surfaces with adsorbed FGF-2

SPR analysis has shown (Fig 5B, Curves 2 and 3) that the amount of FGF-2 adsorbed (FGF-2_ads_) on the (Alb/Hep)_2_ coating can be controlled by varying the time of surface exposition to FGF-2 and the initial FGF-2 concentration in suspending medium. By controlling these two factors, (Alb/Hep)_2_ assemblies were prepared with a lower amount of FGF-2 corresponding to 30 ng of FGF-2/cm^2^ (denoted under the graphs as (Alb/Hep)_2_FGF-2_adsLow_) and a higher amount corresponding to 120 ng of FGF-2/cm^2^ (denoted under the graphs as (Alb/Hep)_2_FGF-2_adsHigh_). To compare the effect of FGF-2_ads_ or FGF-2_sol_, cells were also cultivated on TCPS and (Alb/Hep)_2_ surfaces with FGF-2 added into the cultivation media in a concentration of 10 ng/mL (denoted as TCPS_FGF-2_sol_ and (Alb/Hep)_2_FGF-2_sol_, respectively). This concentration was found to be optimal for stimulating CPAE cell proliferation (S6 Fig). TCPS surfaces with or without FGF-2_sol_ in the media were considered as control surfaces. Most of the cells were spread 24 h after seeding, although their morphology differed (S5 Fig and S8 Fig); on (Alb/Hep)_2_ assemblies with FGF-2_ads_ the cells were more elongated as compared to the cells cultured on TCPS. Similar morphological features were also found in the bovine aortic endothelial cells GM-7373 cultured on the FGF-2-coated plastic surface.[56] The shape of the seeded endothelial cells can depend on the surface roughness of the substrate. The AFM analysis revealed that the presence of FGF-2_ads_ caused a slight increase in the RMS surface roughness of the (Alb/Hep)_2_ assembly (Fig 6). Generally, the roughness of eLbL films increases with the increasing film thickness. Wittmer et al. reported,[57] that the human endothelial cells (HUVEC) lost their circularity proportionally to the increasing number of deposited layers in poly(L-lysine)/dextran sulfate eLbL film. In another study, endothelial cells (ECs) were cultured on flat, nano-rough and sub-micron-rough titanium surfaces.[58] The ECs seeded on sub-micron surfaces with roughness of 10 nm showed high cell aspect ratios (i.e. cell length/cell width) than on flat substrates with roughness around 2 nm. ECs elongation could be influenced by the surface nano-topography as well. ECs seeded onto poly(dimethylsiloxane) films with periodic arrays of nano-grooves (500 nm thick) with spacing ranging from 22 to 80 μm and alternating nano- and micron roughness were more elongated on the surfaces with the widest spacing of nano-grooves.[59]

**Fig 9:**
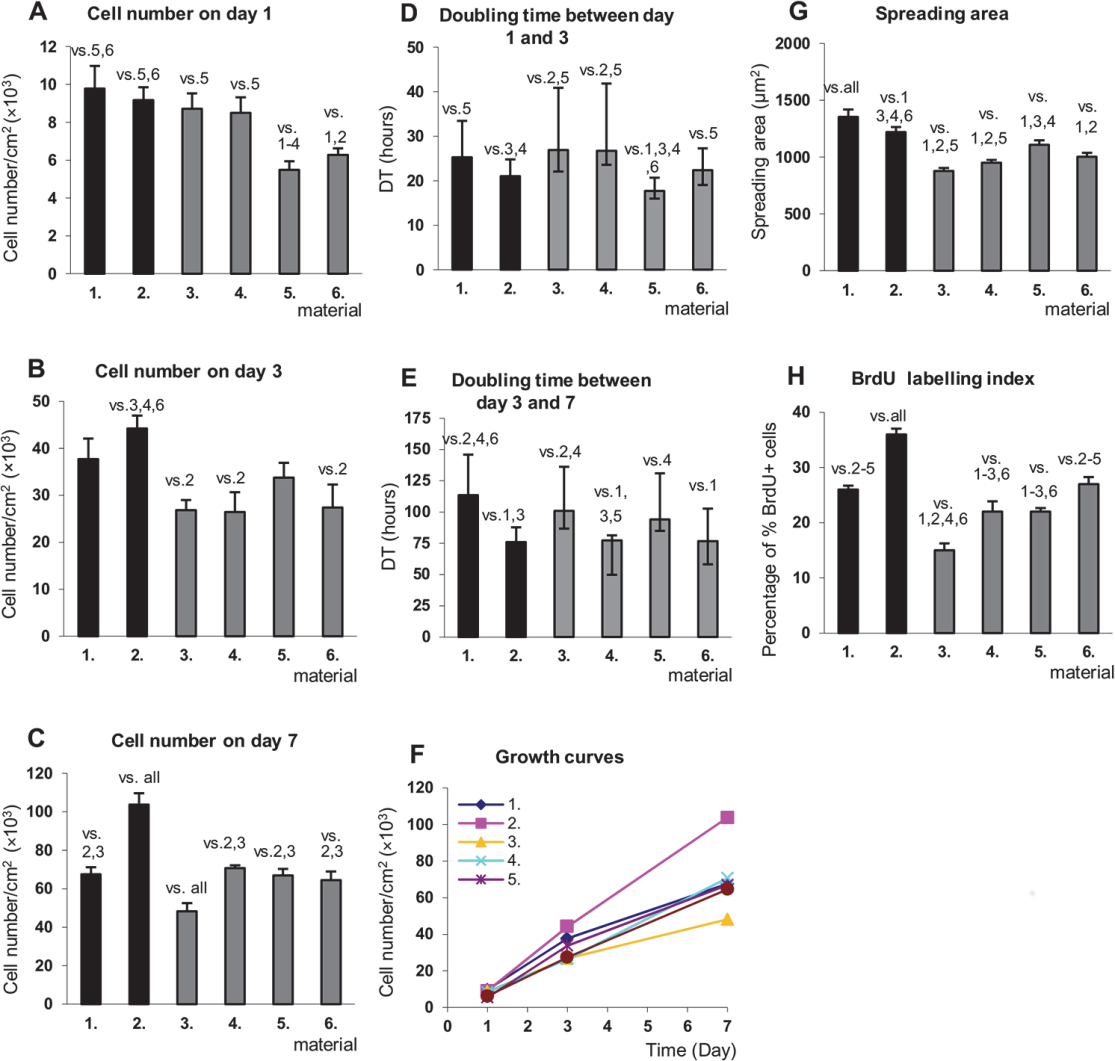
Density of CPAE cells on day 1 (A), 3 (B) and 7 (C), doubling time between day 1 and 3 (D) and between 3 and 7 (E), growth curves (F), cell spreading area on day 1 (G) and BrdU labelling index on day 3 (H) on different surfaces. (**1**) tissue culture polystyrene (TCPS); (**2**) TCPS with 10 ng/mL of FGF-2_sol_ in media (TCPS_FGF-2_sol_); (**3**) (Alb/Hep)_2_; (**4**) (Alb/Hep)_2_ with 10 ng/mL of FGF-2_sol_ in media ((Alb/Hep)_2_FGF-2_sol_); (**5**) (Alb/Hep)_2_ with FGF-2_ads_ adsorbed (30 ng/cm^**2**^, (Alb/Hep)_2_FGF-2_adsLow_); (**6**) (Alb/Hep)_2_ with FGF-2_ads_ adsorbed (120 ng/cm^**2**^, (Alb/Hep)_2_FGF-2_adsHigh_). The cells were cultivated in 5% FBS media. The data is expressed as mean ± S.E.M (A-C, F-H) and median ± quartiles (D, E); Statistically significant differences (p < 0.05) between surfaces (denoted in numbers) are depicted above bars.

On day 1, the cell densities on the (Alb/Hep)_2_ assemblies with the adsorbed FGF-2_ads_ were significantly reduced compared to the other surfaces (Fig 9A). Among (Alb/Hep)_2_ surfaces, the lowest spreading area was on (Alb/Hep)_2_ without any form of FGF-2, while the highest cell spreading area was found on (Alb/Hep)_2_FGF-2_adsLow_ surface (Fig 9G). The mechanisms responsible for adhesion of the endothelial cells to the immobilized FGF-2 were investigated by Rusnati et al.[60] They showed that surface-bound FGF-2 stimulated cell adhesion and spreading of cultured endothelial cells of different origin. This phenomenon was dependent on the interaction of the immobilized FGF-2 with the vitronectin receptor α_v_β_3_.

The most intensive proliferation between day 1 and 3, measured as the doubling time, was on (Alb/Hep)_2_FGF-2_adsLow_ and on TCPS_FGF-2_sol_ (Fig 9D). Slower proliferation was detected on TCPS, (Alb/Hep)_2_ and (Alb/Hep)_2_FGF-2_sol_. On day 3, all (Alb/Hep)_2_ surfaces expressed lower cell densities (Fig 9B) and synthesis of DNA (Fig 9H) than both TCPS controls. However, FGF-2 present in any form improved the BrdU labelling index and supported further cell proliferation between day 3 and 7 (Fig 9C and 9E). On day 7, the cells reached the same density on all (Alb/Hep)_2_ surfaces in the presence of FGF-2 (Fig 9C). Among the (Alb/Hep)_2_ surfaces tested, the (Alb/Hep)_2_FGF-2_adsLow_ assembly seems to support cells growth, especially in the early time intervals. Similarly, the study by Ma et al.[26] showed improved cell proliferation of human gingival fibroblast on titanium with immobilized FGF-2. Almodovar et al.[20] observed that FGF-2 adsorbed on chitosan/heparin films led to higher density of mesenchymal stem cells (MSCs) as compare to FGF-2 added into the culture media. In the study of Bos et al.,[27] primary human endothelial cells (HUVEC) were cultured on albumin, albumin-heparin conjugate and albumin-heparin conjugate coated with fibronectin. Loading the layers with FGF-2 (205 ng/mL) improved the cell growth on day 3 in comparison with the samples with FGF-2 soluble in the medium (0.3 ng/mL). However, the densities on day seven were the same. Comparable cell densities after one week cultivation, irrespective of whether the FGF-2 was adsorbed (FGF-2_ads_) or simply added into the cultivation media (FGF-2_sol_), were also observed in our study (Fig 9C).

**Fig 10:**
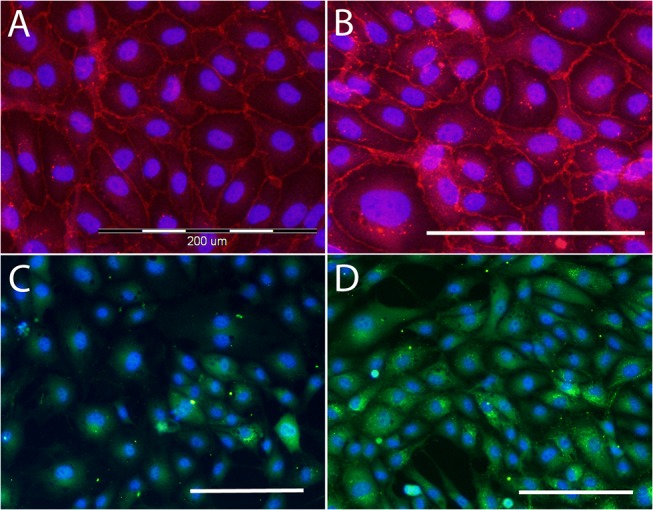
Immunofluorescence staining of VE-cadherin (A,B) and von Willebrand factor (C,D) in CPAE. Cells cultivated on (Alb/Hep)_2_ (A,C) and on (Alb/Hep)_2_ with the adsorbed FGF-2_ads_ (30 ng/cm^**2**^; (Alb/Hep)_2_FGF-2_adsLow_; B,D). The cells are counterstained with Hoechst 33342. Obj. ×20 (A,B), obj. ×10 (C,D), scale bar = 200 μm.

**Fig 11:**
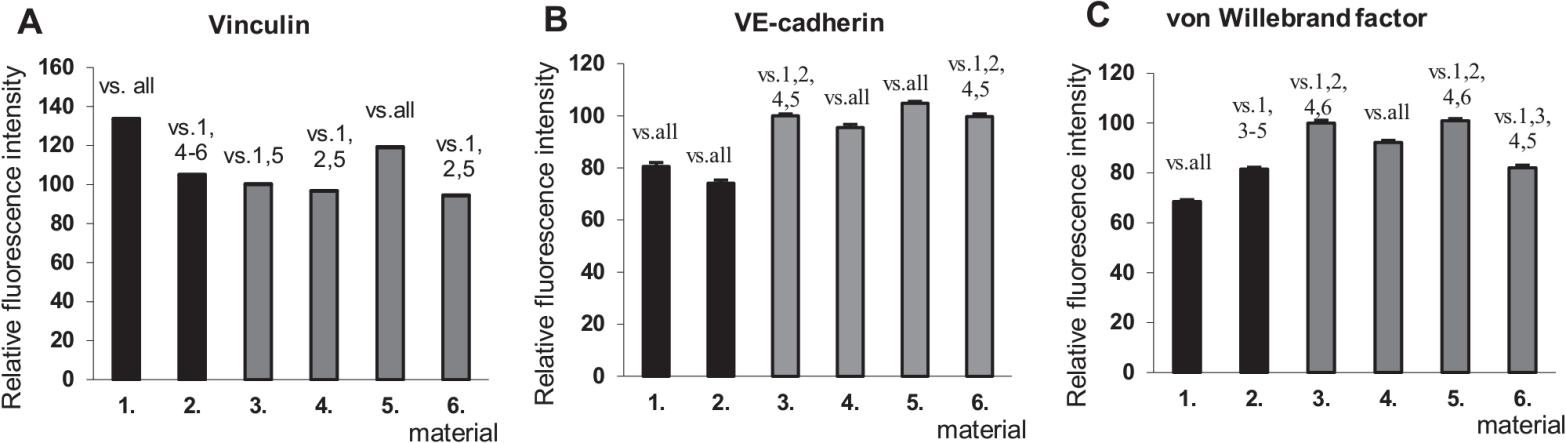
Intensity of immunofluorescence staining for vinculin (on day 3, A), VE-cadherin (on day 7, B) and von Willebrand factor (on day 7, C) of CPAE cells cultured on different surfaces. (**1**) tissue culture polystyrene (TCPS); (**2**) TCPS with 10 ng/mL of FGF-2_sol_ in media (TCPS_FGF-2_sol_); (**3**) (Alb/Hep)_2_; (**4**) (Alb/Hep)_2_ with 10 ng/mL of FGF-2_sol_ in media ((Alb/Hep)_2_FGF-2_sol_); (**5**) (Alb/Hep)_2_ with adsorbed FGF-2_ads_ (30 ng/cm^**2**^, (Alb/Hep)_2_FGF-2_adsLow_); (**6**) (Alb/Hep)_2_ with adsorbed FGF-2_ads_ (120 ng/cm^**2**^, (Alb/Hep)_2_FGF-2_adsHigh_). Cells were cultured in 5% FBS media. Fluorescence intensity is normalized per cell. The data is expressed as mean ± S.E.M. Statistically significant differences (p value <0.05) between surfaces (denoted in numbers) are depicted above bars.

Immunofluorescence staining of VE-cadherin, a specific protein for endothelial cells involved in cell-cell interactions, and von Willebrand factor, a differentiation marker of endothelial cells, were performed on day 7 (Fig 10, S9 Fig and S10 Fig). A more apparent peripheral localization of VE-cadherin was observed on the (Alb/Hep)_2_ surfaces compared to the predominantly diffusional staining on TCPS (S9 Fig). Vascular endothelial cadherin (VE-cadherin) is a major adhesion molecule involved in endothelial junctions,[61] and its stability at junctions is supported by FGF-2 signaling processes regulating the tyrosine phosphatase SHP2 expression.[62] In other words, FGF-2 signaling is highly desirable for the integrity of the endothelial cell monolayer. The FGF-2_ads_ adsorbed on the (Alb/Hep)_2_ coatings significantly supported the formation of adherens junctions between the CPAE cells (Fig 10B). Immunofluorescence staining for VE-cadherin showed that many gaps formed in the CPAE cell monolayer cultured on (Alb/Hep)_2_ and TCPS surfaces with no FGF-2 present (Fig 10A, S9 Fig), while the cell-cell contact was intensified and the gaps were diminished in the presence of FGF-2_ads_ (Fig 10B, S9 Fig). Again, the (Alb/Hep)_2_FGF-2_adsLow_ assembly, showed a significantly higher fluorescence signal for VE-cadherin staining than other coatings (Fig 11B).

Immunofluorescence staining of von Willebrand factor showed the presence of Weibel-Palade bodies in all CPAE cells (S10 Fig). The staining was, however, significantly more intensive on all (Alb/Hep)_2_ surfaces, especially with FGF-2_ads_ adsorbed from a lower concentration (Fig 10C; (Alb/Hep)_2_FGF-2_adsLow_). Our results correlates with previously reported findings that the growth factors can be more effective when attached to the substrate. FGF-2 bound to the cross-linked heparin/chitosan scaffold was more available to the neural stem cells than FGF-2 dissolved in the cell culture medium.[63] Similarly, bone morphogenetic protein 2 immobilized on silk fibroin films stimulated osteogenic differentiation, unlike the soluble factor delivered into the media.[64] When the transforming growth factor-beta1 (TGF-β1) was tethered to the PEG hydrogel,[65] a marked increase in extracellular matrix production by vascular smooth muscle cells in comparison with the same amount of soluble TGF-β1 was detected.

CPAE cells were stained for vinculin, an integrin receptors-associated protein, on day 3. The signal obtained was mostly diffuse, with very small dot-like focal adhesions (data not shown). The fluorescence intensity per cell was significantly higher on TCPS and on (Alb/Hep)_2_FGF-2_adsLow_ (Fig 11A). Interestingly, FGF-2_sol_ had no effect on the vinculin expression. This finding is in accordance with previous observations, where corneal endothelial cells stimulated with FGF-2_sol_ (10 ng/mL in a culture medium) for 3 days demonstrated a loss of stress fibres and focal adhesions.[66] The expression of vinculin was similar for cells maintained with or without FGF-2_sol_, but in response to FGF-2 vinculin was translocated from the focal adhesion complex to the cytoplasm.

The biological studies using a model endothelial cell line CPAE pointed out an importance of the form of FGF-2 delivery and as well as FGF-2 concentration on the overall cellular behavior. Therefore, the eLbl Alb/Hep assembly with a well-controlled loading of FGF-2 developed in this study can help to understand the cell responses induced by bio-signaling molecules. The Alb/Hep assembly also holds a significant potential for loading other than FGF-2 molecules based on electrostatic interactions with a negatively charged heparin, on specific interactions (e.g. heparin-binding proteins) as well as based on a covalent conjugation to functional groups present in both albumin and heparin molecules. Additionally, albumin used as the first anchoring layer can easily adsorb on hydrophobic surfaces (e.g. polystyrene plates) without the need for an additional surface modification in contrast, for example, to widely used poly(L-lysine)/hyaluronan eLbLs.[24]

## Conclusions

We have shown that chemically cross-linked Alb/Hep assemblies prepared by the electrostatic layer-by-layer method can be effectively used for physically immobilizing basic fibroblast growth factor (FGF-2_ads_). Two bilayers of Alb/Hep homogeneously coated a support, and the heparin content in the (Alb/Hep)_2_ assembly was sufficient to immobilize FGF-2. SPR analysis showed that the amount of FGF-2_ads_ on the (Alb/Hep)_2_ assembly can be tuned up to 120 ng/cm^2^ by varying the deposition conditions, i.e. the incubation time of FGF-2 with the (Alb/Hep)_2_ surface and the FGF-2 concentration in the adsorption solution. The FGF-2_ads_ adsorbed on the (Alb/Hep)_2_ assembly retained its bioactivity and stimulated cell proliferation, differentiation, cell spreading, cell-cell interactions and the formation of focal adhesion of CPAE cells in a dose-dependent manner. Despite a smaller initial cell attachment on the FGF-2_ads_ functionalized (Alb/Hep)_2_ surfaces, FGF-2_ads_ enhanced cell proliferation during 7 day cultivation. Further, FGF-2_ads_ adsorbed at a lower concentration of 30 ng/cm^2^ led to significantly improved differentiation and cell-cell interactions in comparison with FGF-2_ads_ adsorbed at a concentration of 120 ng/cm^2^ or FGF-2_sol_ added into the media.

In conclusion, this paper has presented a proposal for a simple method for fabricating eLbL coatings with a controlled FGF-2 surface concentration.

## Supporting Information

S1 Fig FTIR MIRS spectra of Alb, Hep and Alb/Hep assembly on polystyrene. (TIF)

S2 Fig AFM topography images of Alb/Hep assemblies deposited on TCPS taken in PBS: (1) TCPS; (2) (Alb/Hep)_1_; (3) (Alb/Hep)_2_; (4) (Alb/Hep)_3_. Image sizes: left column—5 × 5 μm, right column—1 × 1 μm, a detail framed in the corresponding image in left column. Z-scale: 25 nm.
(TIF)

S3 Fig AFM topography images of the (Alb/Hep)_2_ assemblies: (A) (Alb/Hep)_2_ at pH 4 before film cross-linking; (B) cross-linked (Alb/Hep)_2_ at pH 7.4. Image size: 1 × 1 μm, Z-scale: 25 nm.
(TIF)

S4 Fig The short-term stability of FGF-2_ads_ on (Alb/Hep)_2_ assemblies. (**A**) SPR sensogram of adsorption and desorption of FGF-2_ads_ on/from (Alb/Hep)_2_ assembly; the surface exposed to FGF-2 solution at concentration of (a) 500 ng/mL FGF-2 for 1 h or (b) 1000 ng/mL FGF-2 for 3 h. (**B**) The time course of FGF-2_ads_ desorption from (Alb/Hep)_2_ assembly into PBS (pH 7.4); the FGF-2_ads_ surface concentration was (a) 30 ng/cm^2^ or (b) 120 ng/cm^2^.
(TIF)

S5 Fig Images of living CPAE cells on (Alb/Hep)_2_ surfaces with different dose of FGF-2_sol_ added into the culture media; the control tissue culture polystyrene (A), (Alb/Hep)_2_ (B), (Alb/Hep)_2_ with 0.1 ng/mL FGF-2_sol_ (C), (Alb/Hep)_2_ with 1 ng/mL FGF-2_sol_ (D), (Alb/Hep)_2_ with 10 ng/mL FGF-2_sol_ (E), (Alb/Hep)_2_ with 100 ng/mL FGF-2_sol_ (F). The cells were cultivated in 5% FBS media. Images taken 24 h after seeding. Obj. ×10, scale bar = 200 μm.
(TIF)

S6 Fig The effect of the amount of FGF-2_sol_ added into the cultivation media on the density of CPAE cells. The cell density on the (Alb/Hep)_2_ surface and the control TCPS one, three and six days after seeding. The amount of the added FGF-2_sol_ to low-serum media (5% FBS) was 0, 0.1, 1, 10, and 100 ng/mL. The data is expressed as mean ± S.E.M; p value <0.05 was considered significant. Statistically significant differences between the samples are depicted above the bars in comparison with 0 ng/mL (*), 0.1 ng/mL (&), 1 ng/mL (**), 10 ng/mL (***), 100 ng/mL (#), and TCPS ($).
(TIF)

S7 Fig Immunofluorescence staining of von Willebrand factor in CPAE cells. The cells cultured on the control TCPS (**A**), (Alb/Hep)_2_ (**B**), (Alb/Hep)_2_ with 0.1 ng/mL FGF-2_sol_ (**C**), (Alb/Hep)_2_ with 1 ng/mL FGF-2_sol_ (**D**), (Alb/Hep)_2_ with 10 ng/mL FGF-2_sol_ (**E**), and on (Alb/Hep)_2_ with 100 ng/mL FGF-2_sol_ (**F**) 6 days after seeding. The cells were cultivated in 5% FBS media. Obj. ×10, scale bar = 200 μm.
(TIF)

S8 Fig CPAE cells stained with Texas Red C_2_ maleimide and Hoechst 33342 on different surfaces. CPAE cells cultured on the control TCPS (**A**), on TCPS with 10 ng/mL of FGF-2_sol_ in media (TCPS_FGF-2_sol_, **B**), on (Alb/Hep)_2_ (**C**), (Alb/Hep)_2_ with 10 ng/mL of FGF-2_sol_ in media ((Alb/Hep)_2_FGF-2_sol_, **D**), (Alb/Hep)_2_ with FGF-2_ads_ adsorbed (30 ng/cm^2^, (Alb/Hep)_2_FGF-2_adsLow_, **E**), and on (Alb/Hep)_2_ with FGF-2_ads_ adsorbed (120 ng/cm^2^, (Alb/Hep)_2_FGF-2_adsHigh_, **F**) 24 h after seeding. The cells were cultivated in 5% FBS media. Obj. ×20, scale bar = 100 μm.
(TIF)

S9 Fig Immunofluorescence staining of VE-cadherin in CPAE on different surfaces. The cells cultured on the tissue culture polystyrene (TCPS, **A**), on TCPS with 10 ng/mL of FGF-2_sol_ in media (TCPS_FGF-2_sol_, **B**), on (Alb/Hep)_2_ (**C**), on (Alb/Hep)_2_ with 10 ng/mL of FGF-2_sol_ in media ((Alb/Hep)_2_FGF-2_sol_, **D**), on (Alb/Hep)_2_ with adsorbed FGF-2_ads_ (30 ng/cm^2^, (Alb/Hep)_2_FGF-2_adsLow_, **E**), and on (Alb/Hep)_2_ with adsorbed FGF-2_ads_ (120 ng/cm^2^, (Alb/Hep)_2_FGF-2_adsHigh_, **F**). The cells are counterstained with Hoechst 33342. Obj. ×20, scale bar = 200 μm.
(TIF)

S10 Fig Immunofluorescence staining of von Willebrand factor in CPAE on different surfaces. The cells cultured on the tissue culture polystyrene (TCPS, **A**), on TCPS with 10 ng/mL of FGF-2_sol_ in media (TCPS_FGF-2_sol_, **B**), on (Alb/Hep)_2_ (**C**), on (Alb/Hep)_2_ with 10 ng/mL of FGF-2_sol_ in media ((Alb/Hep)_2_FGF-2_sol_, **D**), on (Alb/Hep)_2_ with adsorbed FGF-2_ads_ (30 ng/cm^2^, (Alb/Hep)_2_FGF-2_adsLow_, **E**), and on (Alb/Hep)_2_ adsorbed FGF-2_ads_ (120 ng/cm^2^, (Alb/Hep)_2_FGF-2_adsHigh_, **F**). The cells are counterstained with Hoechst 33342. Obj. ×10, scale bar = 200 μm.
(TIF)
