# Supplementary figures and images for: Cellular Responses Modulated by FGF-2 Adsorbed on Albumin/Heparin Layer-by-Layer Assemblies
Source: PLoS One. 2015 May 6;10(5):e0125484. doi: 10.1371/journal.pone.0125484 (PMC4422587; doi:10.1371/journal.pone.0125484)

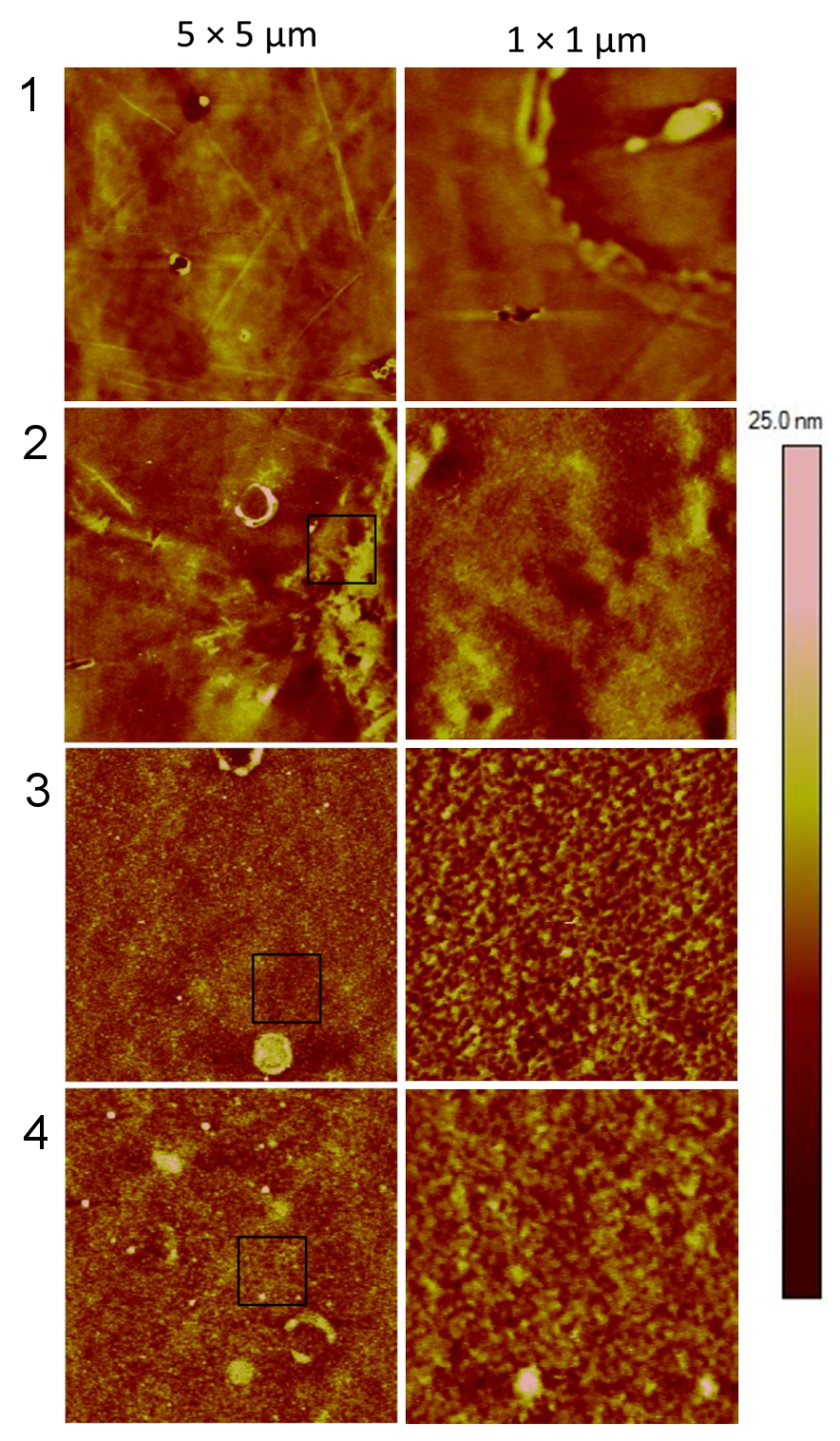

Supplement: S2 Fig — Image sizes: left column—5 × 5 μm, right column—1 × 1 μm, a detail framed in the corresponding image in left column. Z-scale: 25 nm. (TIF) [file pone.0125484.s002.tif]

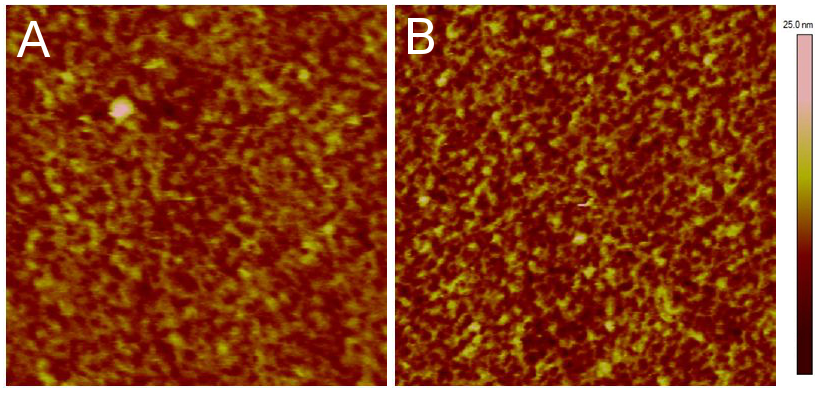

Supplement: S3 Fig — Image size: 1 × 1 μm, Z-scale: 25 nm. (TIF) [file pone.0125484.s003.tif]

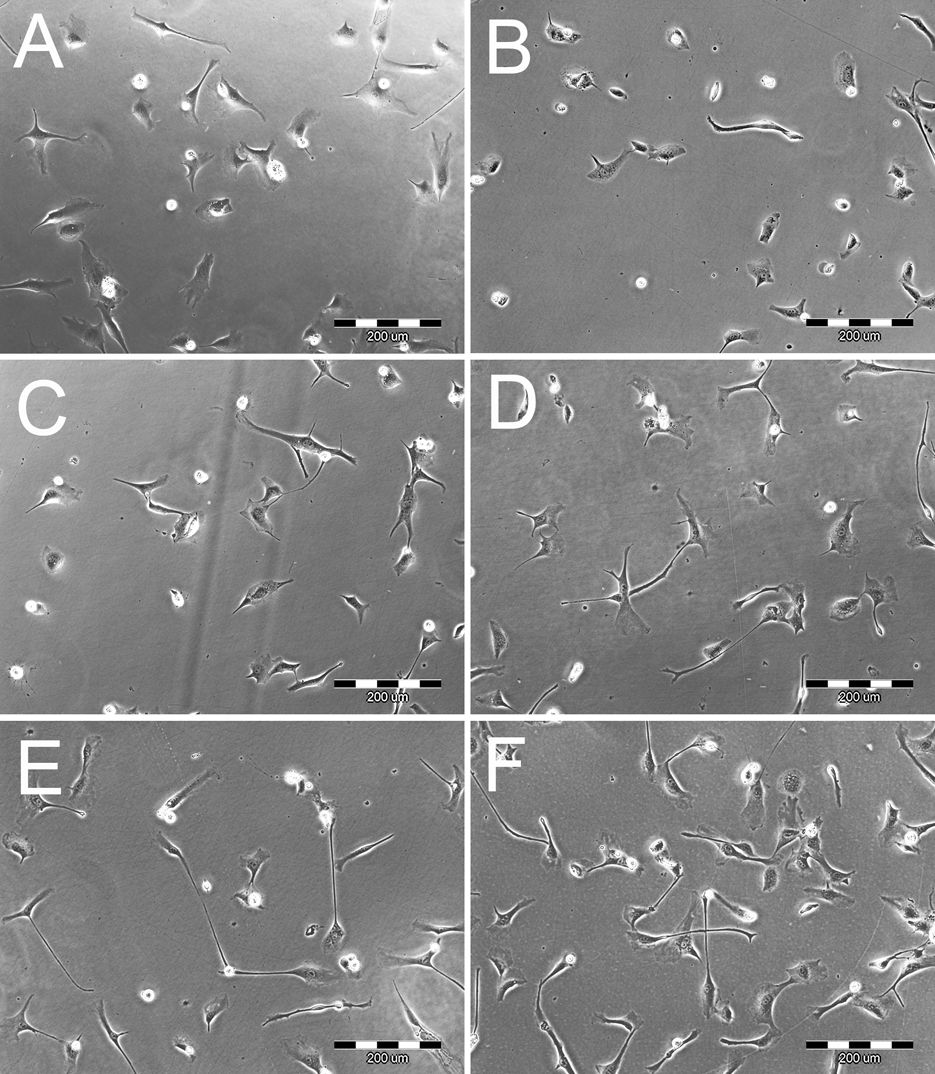

Supplement: S5 Fig — The cells were cultivated in 5% FBS media. Images taken 24 h after seeding. Obj. ×10, scale bar = 200 μm. (TIF) [file pone.0125484.s005.tif]

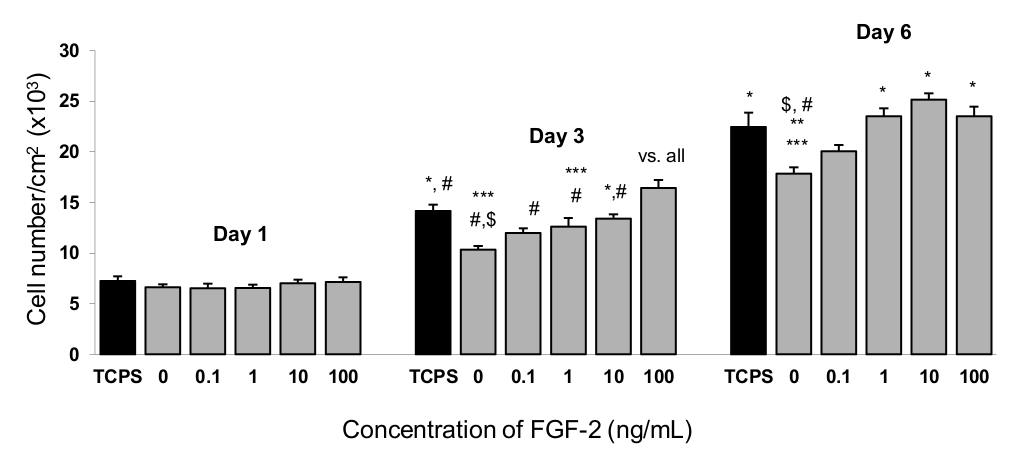

Supplement: S6 Fig — The cell density on the (Alb/Hep)2 surface and the control TCPS one, three and six days after seeding. The amount of the added FGF-2sol to low-serum media (5% FBS) was 0, 0.1, 1, 10, and 100 ng/mL. The data is expressed as mean ± S.E.M; p value <0.05 was considered significant. Statistically significant differences between the samples are depicted above the bars in comparison with 0 ng/mL (*), 0.1 ng/mL (&), 1 ng/mL (**), 10 ng/mL (***), 100 ng/mL (#), and TCPS ($). (TIF) [file pone.0125484.s006.tif]

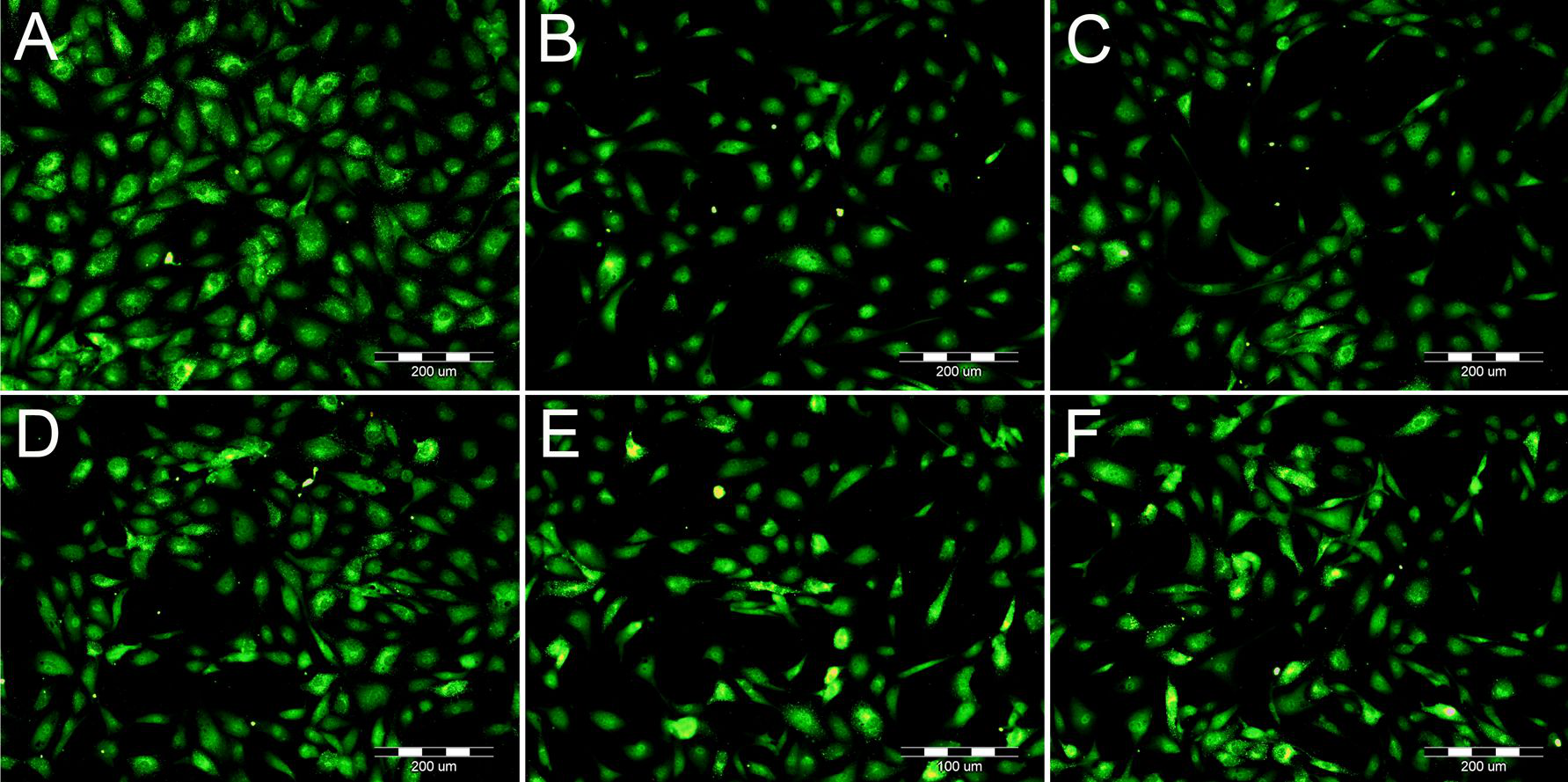

Supplement: S7 Fig — The cells cultured on the control TCPS (A), (Alb/Hep)2 (B), (Alb/Hep)2 with 0.1 ng/mL FGF-2sol (C), (Alb/Hep)2 with 1 ng/mL FGF-2sol (D), (Alb/Hep)2 with 10 ng/mL FGF-2sol (E), and on (Alb/Hep)2 with 100 ng/mL FGF-2sol (F) 6 days after seeding. The cells were cultivated in 5% FBS media. Obj. ×10, scale bar = 200 μm. (TIF) [file pone.0125484.s007.tif]

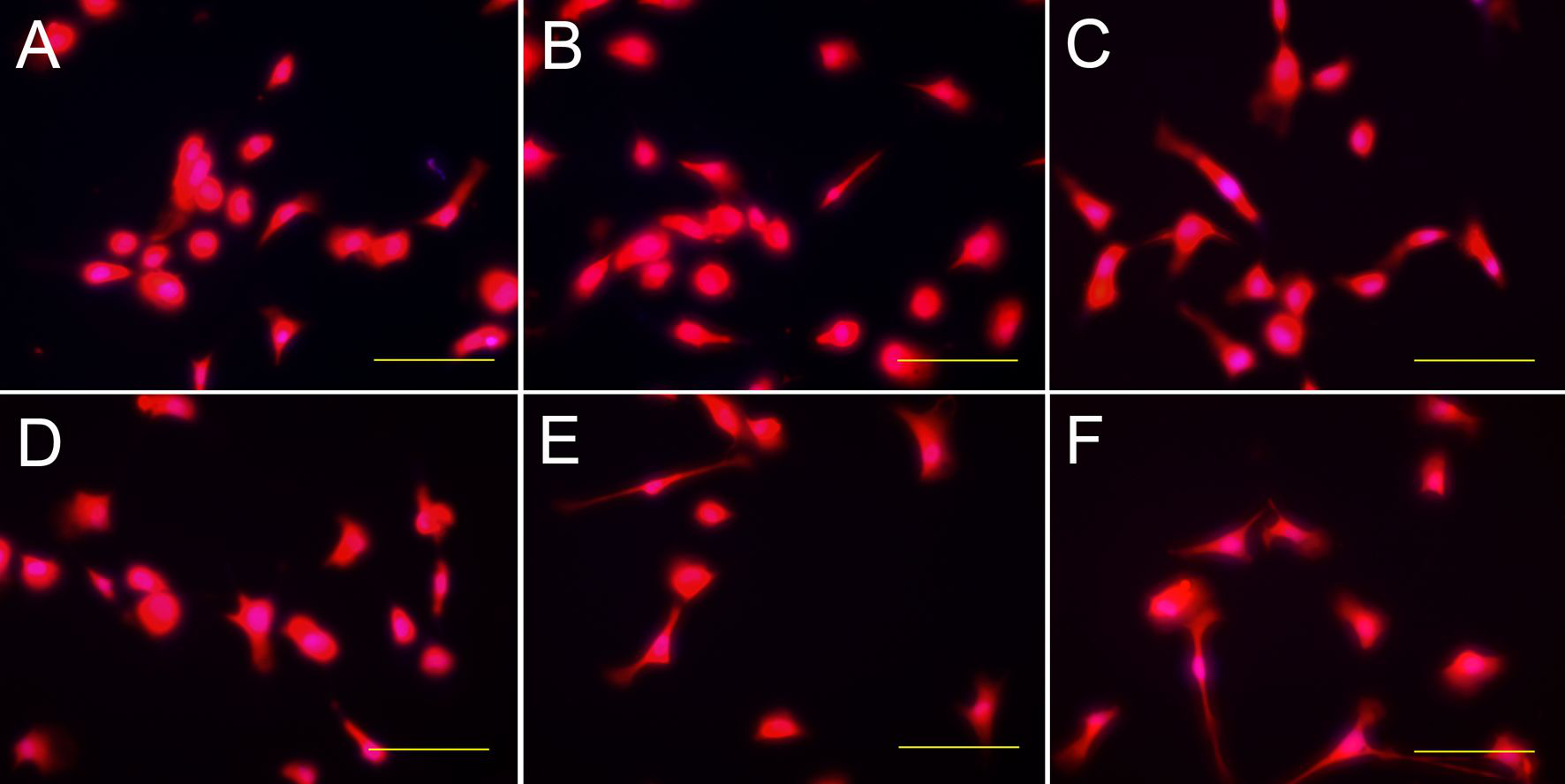

Supplement: S8 Fig — CPAE cells cultured on the control TCPS (A), on TCPS with 10 ng/mL of FGF-2sol in media (TCPS_FGF-2sol, B), on (Alb/Hep)2 (C), (Alb/Hep)2 with 10 ng/mL of FGF-2sol in media ((Alb/Hep)2FGF-2sol, D), (Alb/Hep)2 with FGF-2ads adsorbed (30 ng/cm2, (Alb/Hep)2FGF-2adsLow, E), and on (Alb/Hep)2 with FGF-2ads adsorbed (120 ng/cm2, (Alb/Hep)2FGF-2adsHigh, F) 24 h after seeding. The cells were cultivated in 5% FBS media. Obj. ×20, scale bar = 100 μm. (TIF) [file pone.0125484.s008.tif]

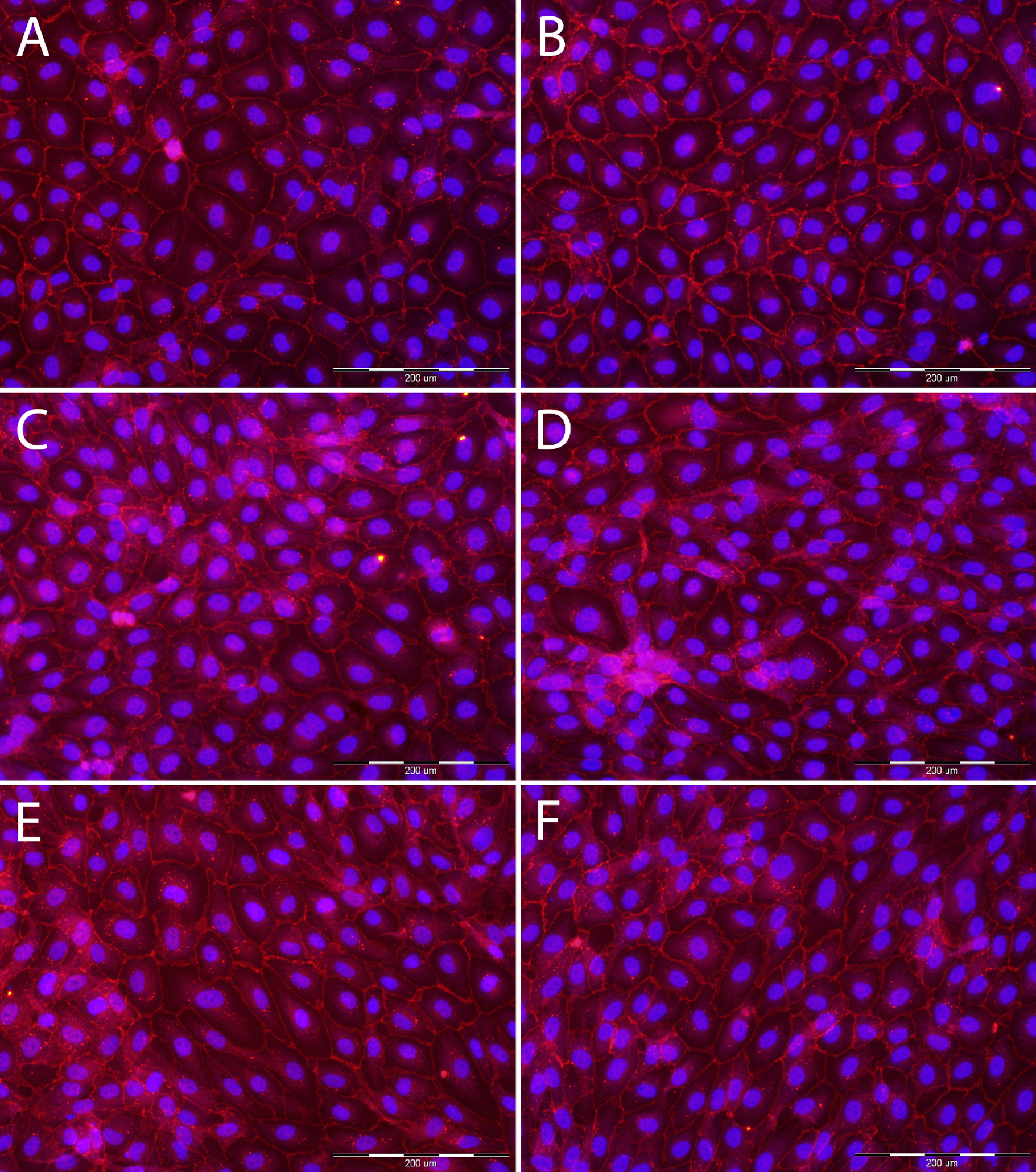

Supplement: S9 Fig — The cells cultured on the tissue culture polystyrene (TCPS, A), on TCPS with 10 ng/mL of FGF-2sol in media (TCPS_FGF-2sol, B), on (Alb/Hep)2 (C), on (Alb/Hep)2 with 10 ng/mL of FGF-2sol in media ((Alb/Hep)2FGF-2sol, D), on (Alb/Hep)2 with adsorbed FGF-2ads (30 ng/cm2, (Alb/Hep)2FGF-2adsLow, E), and on (Alb/Hep)2 with adsorbed FGF-2ads (120 ng/cm2, (Alb/Hep)2FGF-2adsHigh, F). The cells are counterstained with Hoechst 33342. Obj. ×20, scale bar = 200 μm. (TIF) [file pone.0125484.s009.tif]

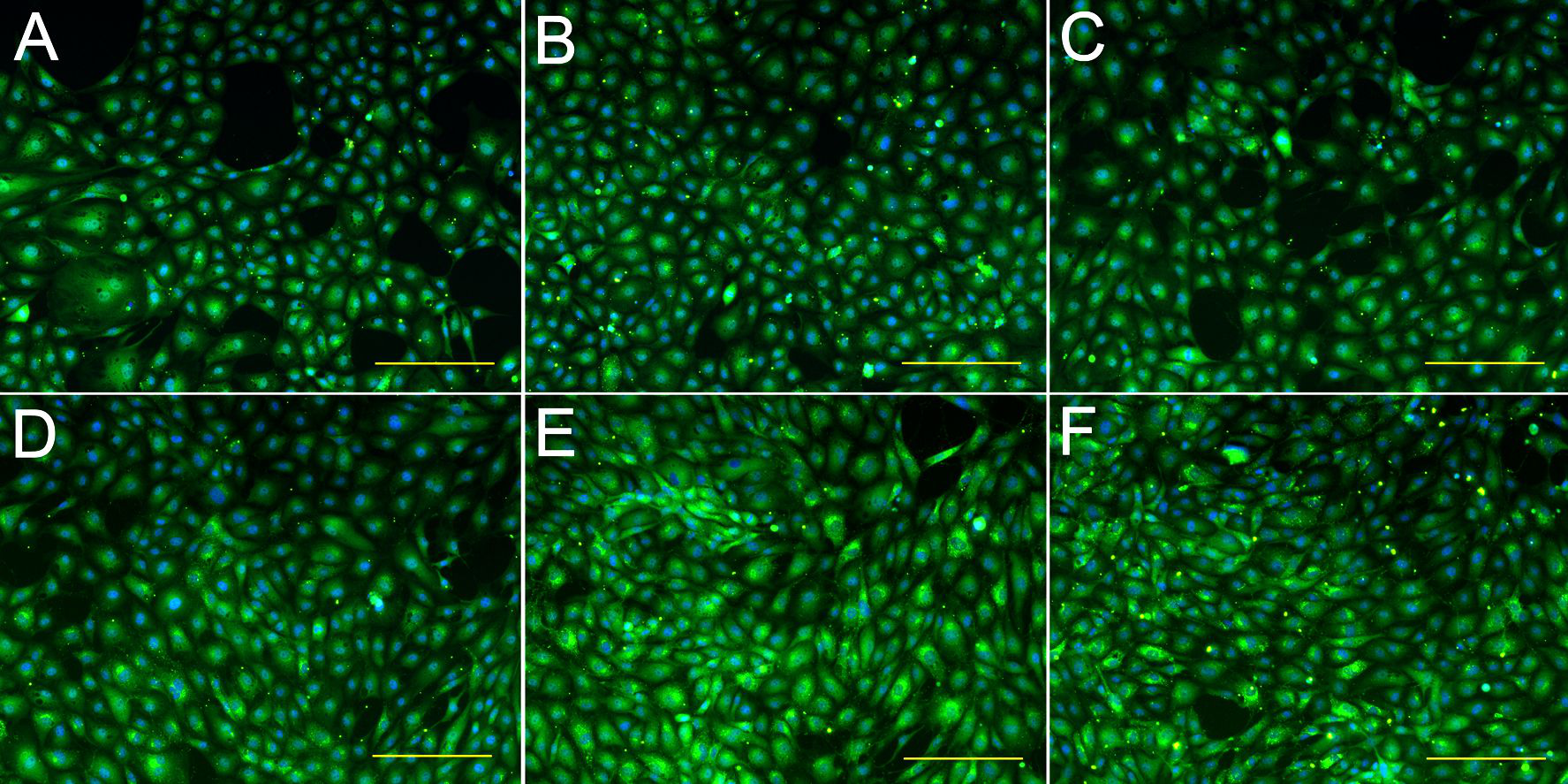

Supplement: S10 Fig — The cells cultured on the tissue culture polystyrene (TCPS, A), on TCPS with 10 ng/mL of FGF-2sol in media (TCPS_FGF-2sol, B), on (Alb/Hep)2 (C), on (Alb/Hep)2 with 10 ng/mL of FGF-2sol in media ((Alb/Hep)2FGF-2sol, D), on (Alb/Hep)2 with adsorbed FGF-2ads (30 ng/cm2, (Alb/Hep)2FGF-2adsLow, E), and on (Alb/Hep)2 adsorbed FGF-2ads (120 ng/cm2, (Alb/Hep)2FGF-2adsHigh, F). The cells are counterstained with Hoechst 33342. Obj. ×10, scale bar = 200 μm. (TIF) [file pone.0125484.s010.tif]
